# Electronic Structure of the Ground and Low-Lying States of MoLi

**DOI:** 10.3390/molecules30132874

**Published:** 2025-07-06

**Authors:** Constantinos Demetriou, Demeter Tzeli

**Affiliations:** 1 Laboratory of Physical Chemistry, Department of Chemistry, National and Kapodistrian University of Athens, 15772 Athens, Greece; costasdim@chem.uoa.gr; 2 Theoretical and Physical Chemistry Institute, National Hellenic Research Foundation, 11635 Athens, Greece

**Keywords:** diatomic molecule, multi-reference configuration interaction, MoLi, chemical bonding, electronic structure

## Abstract

Molybdenum lithium compounds and materials are being researched and applied in cutting-edge industries; however, their bonding has not been explored in a systematic way. The present study investigates the MoLi molecule, to shed light on its bonding. Specifically, the electronic structure and bonding of the ground and 40 low-lying states of the MoLi molecule are explored, employing multireference methodologies, i.e., CASSCF and MRCISD(+Q) in conjunction with the aug-cc-pV5z(-PP) basis set. Bond distances, dissociation energies, dipole moments as well as common spectroscopic constants are given, while the potential energy curves are plotted. For the ground state, XΣ+6, it is found that $R_{e}$ = 2.708 Å, $D_{e}$ = 24.1 kcal/mol, $\omega_{e}$ = 316.8 ${\mathrm{cm}}^{-1}$, $\omega_{e}x_{e}$ = 2.11 ${\mathrm{cm}}^{-1}$, and *μ* = 3.63 D. Overall, the calculated states present a variety of bonds, from weak van der Waals up to the formation of 2.5 bonds. The dissociation energies of the calculated states range from 2.3 kcal/mol (aΣ+8) to 34.7 (cΠ4), while the bond distances range from 2.513 Å to 3.354 Å. Finally, dipole moment values up to 3.72 D are calculated. In most states, a 2s${2p}_{z}$ hybridization on Li and a ${4d}_{z^{2}}$5s${5p}_{z}$ or 5s${5p}_{z}$ hybridization on Mo are found. Moreover, it is observed that the excited Li(P2) atom forms the shortest bonds because its empty ${2s}^{0}$ orbital can easily accept electrons, resulting in a strong σ dative bond. Finally, the present work highlights the exceptional ability of lithium atoms to participate in a variety of bonding schemes, and it could provide the opening gate for further investigation of this species or associated material and complexes.

## 1. Introduction

Molybdenum lithium materials have specialized applications across several industries due to the unique physical and chemical properties of both elements [1,2,3]. Specifically, the use of molybdenum in lithium-based applications has promising roles in energy storage, battery technologies, lubrication, catalysis, electronics, etc. [4,5,6,7,8]. Furthermore, materials or compounds, including Mo-Li bonds, are being researched and applied in cutting-edge industries, like electric vehicles, renewable energy systems, and advanced manufacturing [9,10,11,12,13]. However, the molecular and electronic structure of the ground and excited states of the molybdenum lithium diatomic molecule have not been investigated, while their bonding has not been explored.

While compounds containing transition metal atoms are of interest, their accurate description on a theoretical level poses a demanding task due to their computational complexity rising from their high density of states and the high space-spin angular momentum of the contained transition metal atoms. Therefore, the understanding of the bonding schemes between a transition metal element and a main group element is not an easy task [14]. Thorough insight into the simplest building blocks of such compounds is the steppingstone towards the investigation of more complex systems [15]. For instance, in the case of the MoS_2_ 2D material, it was found that the low-lying septet states of the diatomic MoS [16] and triatomic MoS_2_ molecules are involved in the material as a building block, explaining the variety of its morphologies [15].

The exploration of the electronic structure of molybdenum compounds is of considerable interest because they present a key role in molecular biology, specifically in nitrogen fixation and oxidation catalysis [17,18]. Interestingly, molybdenum (Mo) has a unique electronic configuration as an element, i.e., all of its valance shells are half filled, Mo: [Kr]${4d}^{5}{5s}^{1}$, allowing it to participate in a multitude of different bonding schemes, with some of them resulting in significant molecular properties [19,20,21,22,23]. Per se, many molybdenum compounds portray an important role as selective catalysts in organic synthesis and in the chemical industry. Lithium (Li), on the other hand, has another interesting electronic configuration on its own, one of a half-filled valence subshell containing a single electron, Li: [He]${2s}^{1}$. With such a simple structure, one would expect Li to exhibit a very blunt chemistry, but it showcases some interesting behaviors [24,25,26], where Li forms double bonds [24].

Until now, most diatomic molecules of molybdenum have been studied to much extent, but this is not the case for the MoLi molecule. As far as we know, there are not any experimental studies on the MoLi molecule. Theoretically, recently, our group studied the ground states of the MoX molecules, where X = Li, Be, B, C, O, N, F via multireference and coupled cluster methodologies, where the correlation energy of both core + valence electrons has been calculated [27]. In the present study, the electronic spectrum of MoLi is investigated for the first time. Specifically, the ground state and 40 low-lying states of the MoLi molecule have been investigated via a complete active space self-consistent field (CASSCF), while 12 of them were further investigated via a multi-reference configuration interaction (MRCISD and MRCISD(Q)). The present study aims to deliver a more detailed discussion of the properties of this molecule, with high precision calculations and to fill the gap in the study of the diatomic molecules. Furthermore, the selected calculated states are correlated with material or complexes that include Mo-Li bonds in their structure.

## 2. Results and Discussion

### 2.1. SA-CASSCF

Given that there was not any previous experimental or theoretical study on the electronic spectra of MoLi, some preliminary SA-CASSCF calculations were carried out, simply for the purpose of mapping the abundance and compactness of the MoLi’s molecular electronic spectrum, from which it was possible to identify 41 electronic states. From the combination of Mo + Li, 41 Λ2S+1 molecular states were gathered at the SA-CASSCF/aug-cc-pV5Z(-PP) computational level, specifically 11 octet states, i.e., (Σ+{3}8,Σ−,Π{3},Δ{3},Φ), 11 sextet states, i.e.,(Σ+{3}6,Σ−,Π{3},Δ{2},Φ,Γ), 9 quartet states, i.e., (Σ+4,Σ−,Π{3},Δ{2},Φ,Γ), and 10 doublet states, i.e., (Σ+{2}2,Π{2},Δ{2}, Φ,Γ,H,Ι). The number in braces, i.e., Π{3}, denotes that three Π states have been calculated. To sum up, all states that adiabatically correlated to Mo(a^7^S, a^5^S, a^5^D) + Li(a^2^S), Mo(a^7^S) + Li(a^2^P) and some states that adiabatically correlated to Mo(a^5^G, a^5^P, a^3^D, a^3^G) + Li(a^2^S, a^2^P) have been calculated. Note that the triplet excited states of Mo, i.e., a^3^P, a^3^D, a^3^G, are very close lying. Regarding, their lowest energy j term, their ordering is a^3^P, a^3^D, a^3^G; however, for their j-average term, the a^3^G term is the lowest one, and this is the reason why the doublet states of MoLi correlates adiabatically to the a^3^G term.

**Figure 1:**
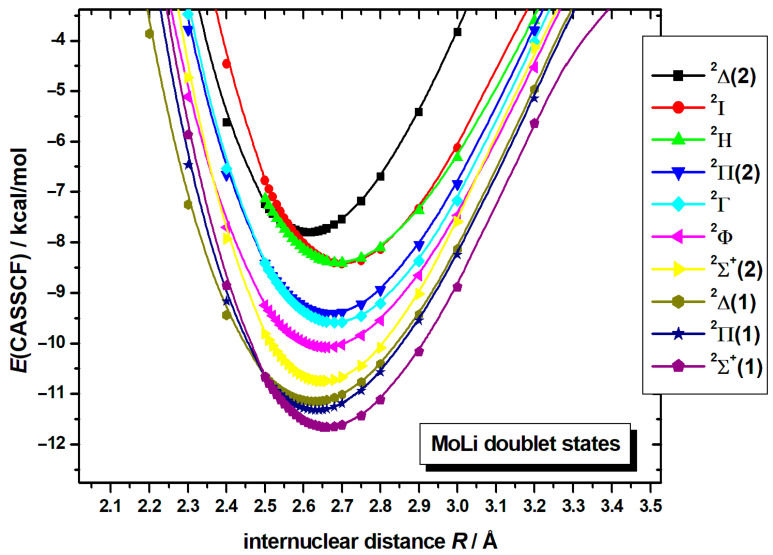
Potential energy curves of the lowest ten doublet states of MoLi at the SA-CASSCF/aug-cc-pV5Z(-PP) computational level. The Mo (a^3^G) + Li (^2^S) limit, to which the ^2^Σ^+^ state correlates, is used to define the zero of energy for this figure.

**Figure 2:**
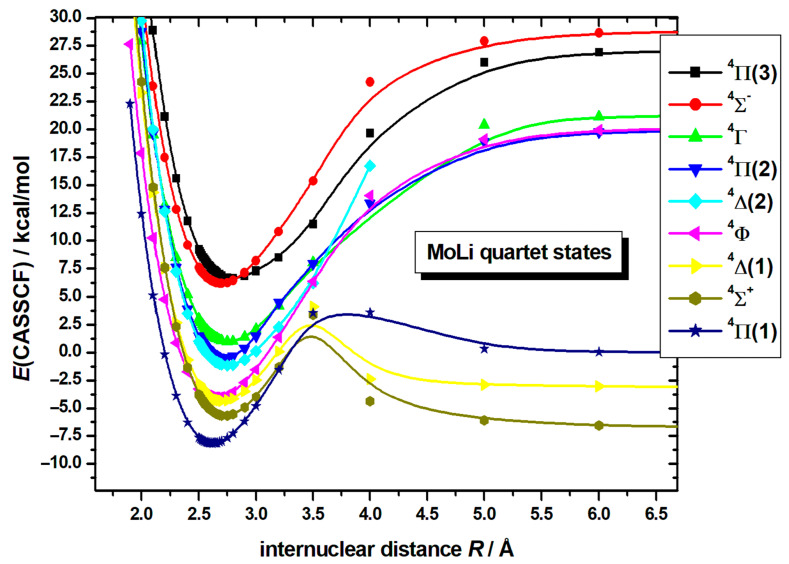
Potential energy curves of the lowest nine quartet states of MoLi at the SA-CASSCF/aug-cc-pV5Z(-PP) computational level. The Mo (a ^5^D) + Li (^2^S) limit, to which the ^4^Π state correlates, is used to define the zero of energy for this figure.

**Figure 3:**
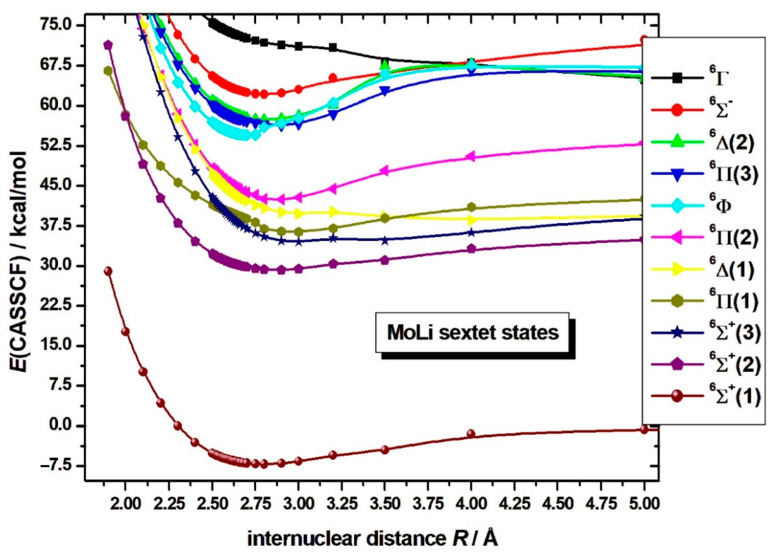
Potential energy curves of the lowest eleven sextet states of MoLi at the SA-CASSCF/aug-cc-pV5Z(-PP) computational level. The Mo (a ^7^S) + Li (^2^S) limit, to which the X ^6^Σ^+^ state correlates, is used to define the zero of energy for this figure.

**Figure 4:**
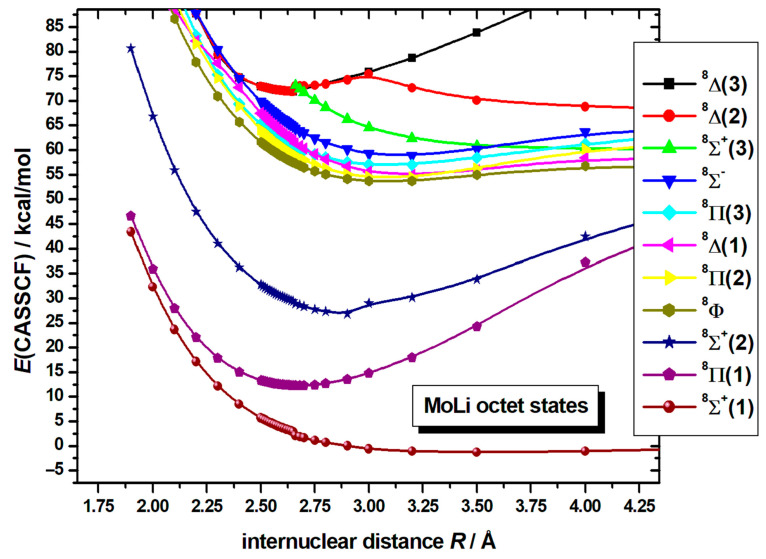
Potential energy curves of the lowest eleven octet states of MoLi at the SA-CASSCF/aug-cc-pV5Z(-PP) computational level. The Mo (a ^7^S) + Li (^2^S) limit, to which the ^8^Σ^+^ state correlates, is used to define the zero of energy for this figure.

The SA-CASSCF potential energy curves for all calculated molecular states are depicted in Figure 1, Figure 2, Figure 3 and Figure 4, their bond distances and the relative energy differences are given in Table 1. In cases where more than one state with the same spin and angular momentum have been calculated, the ordering is given in parenthesis, i.e., ^4^Π(2) is the second ^4^Π state. The bond distances range from 2.61 Å to 3.22 Å, while three of the calculated states are repulsive at the CASSCF level of theory, i.e., ^8^Σ^+^(1), ^8^Σ^+^(3), and ^6^Γ. The shortest bond distances are observed for the doublet and quartet states, i.e., ^2^Δ(2), ^4^Π(1), ^2^Δ(1), and ^2^Π(1), see Table 1.

The PECs of the doublet states have been plotted in Figure 1. They are lying about 70 kcal/mol (=3.1 eV) above the ground state, and they present bond distances around 3.1 Å, see Table 1. Quartet states are lying about 42 kcal/mol (=1.8 eV) above the ground state. Their PECs are plotted in Figure 2, where it is clearly shown that the three lowest in energy quartet states, i.e., ^4^Π(1), ^4^Σ^+^, and ^4^Δ(1), present avoided crossing at about 3.5 Å with the ^4^Π(2), ^4^Σ^+^(2), and ^4^Δ(2) excited states, respectively, see discussion below. The eleven lowest in energy sextet states are plotted in Figure 3. It is found that the ground state is the X ^6^Σ^+^ state, which is separated clearly from the remaining sextet states, i.e., the X state is about 36 kcal/mol (=1.6 eV), lying below their bunch of states. Avoided crossings are observed between the ^6^Π(1) the ^6^Π(2) states. Moreover, the ^6^Φ and ^6^Δ(2) states present crossing with higher in energy states that have not been calculated; however, the crossings are observed in their configurations and depicted in Figure 3. Finally, the octet states are plotted in Figure 4. SA-CASSCF and CASSCF methods predict that the ^8^Σ^+^ is repulsive; however, MRCISD and MRCISD+Q confirm that the state is slightly bound. It is a van der Waals state, see discussion below. The excited ^8^Δ(2) and ^8^Δ(3) states present avoided crossing at about 3.0 Å. It is quite interesting that three quartet states, i.e., ^8^Π, ^8^Δ(2), and ^8^Δ(3) present short bond distances of about 2.66 Å, even though the states present seven single occupied orbitals, see discussion below.

### 2.2. MRCISD(+Q) Methods

**Figure 5:**
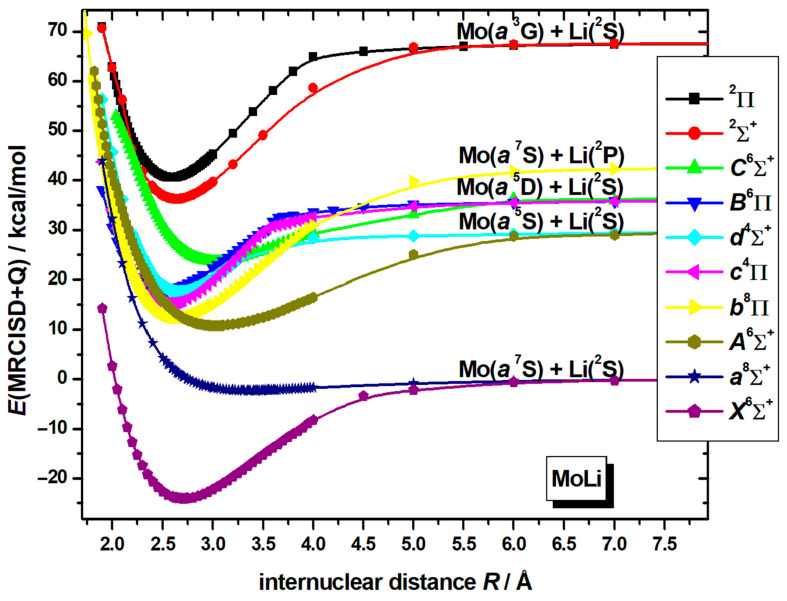
PECs of the selected states of MoLi at the MRCISD+Q/aug-cc-pV5Z(-PP) computational level. The Mo (a ^7^S) + Li (^2^S) limit, to which the X^6^Σ^+^ state correlates, is used to define the zero of energy for this figure.

Based on their relative energetic positions, sequential order, and angular symmetry characteristics, the first electronic state of each spin multiplicity exhibiting Σ and Π symmetries—within the $C_{\infty v}$ point group of the molecule—was identified, under the assumption that these symmetries are the most relevant for molecular bonding, in light of the atomic electronic configurations, i.e., the eight electronic states XΣ+6(1), Π6(1), Σ+8(1), Π8(1), Π4(1), Σ+4(1), Σ+2(1), and Π2(1). Furthermore, the two excited states that lay very close to the X state and portray the same spin and angular symmetry, i.e., Σ+6(2), and Σ+6(3), as well as two states that present avoided crossing, i.e., Π4(2), with the Π4(1) state, and Π6(2), with the Π6(1) state, respectively, were further investigated at the MRCISD(+Q) level of theory, for a more accurate description. As such, CASSCF calculation—providing a multi-reference wavefunction that captures static correlation and optimized orbitals—preceded the subsequent MRCISD for each state, which were performed in conjunction with an augmented, correlation-consistent, polarized valence basis set of quintuple-ζ quality. The PECs of these states were calculated, see Figure 5 and Figure S1 of Supplementary Materials, while their bonding across their PECs were analyzed. It is noteworthy that while the Σ+63 state of MoLi is hanging lower than the Π61 at CASSCF/aug-cc-pV5Z(-PP) level of theory, it was found that the order is reversed at the MRCISD and MRCISD+Q/ aug-cc-pV5Z(-PP) computational levels. Additionally, the ordering of the Σ+62 and Π81 states changes from CASSCF to MRCISD, and finally in MRCISD+Q, i.e., at CASSCF, the Π81 is lower in energy than the Σ+62 state. At MRCISD, the two states are energetically degenerate, and finally, at MRCISD+Q, the Σ+62 state is the lowest between these two states, cf Table 1 and Table 2. It should be noted that CASSCF and SA-CASSCF present the same relative energy ordering of the states in general; however, when the states differ about 3 kcal/mol or less, their relative energy ordering may be altered.

To sum up, our best results are obtained at the MRCISD+Q level. Thus, the states are named according to their MRCISD+Q energies as XΣ+6, aΣ+8, AΣ+62, bΠ8, cΠ4, dΣ+4, BΠ6, and CΣ+63, Π62, Π42, Σ+2, and Π2. For these twelve electronic states, their usual spectroscopic parameters, such as bond distances, dissociation energies, dipole moments, harmonic and anharmonic corrections, and relative adiabatic energies at CASSCF, MRCISD and MRCISD+Q/aug-cc-pV5Z(-PP) are provided in Table 2, and their leading equilibrium CASSCF configuration accompanied by its corresponding molecular orbitals’ are given in Table 3. Over the following paragraphs, we further discuss in detail the bonding of selected electronic states.

#### 2.2.1. XΣ+6

The XΣ+6 state is clearly the ground one, see Table 2. It correlates to the Mo(aS7) + Li(S2) adiabatic atomic products, and these atomic states are involved in the bonding. The CASSCF leading configuration in equilibrium, followed by the Mulliken atomic distributions (Mo/Li) are: |XΣ6+≅ 0.92 $\left.|{1\sigma }^{2}{2\sigma }^{1}{1\delta }_{+}^{1}{1\pi }_{x}^{1}1\pi_{y}^{1}1\delta_{-}^{1}\right\rangle$

$$5s^{1.29}5p_{z}^{0.07}5p_{x}^{0.03}5p_{y}^{0.03}4d_{z^{2}}^{0.97}4d_{x^{2}-y^{2}}^{0.98}4d_{xz}^{0.96}4d_{yz}^{0.96}4d_{xy}^{0.98}/2s^{0.55}2p_{z}^{0.04}2p_{x}^{0.03}2p_{y}^{0.03}$$

**Scheme 1:**
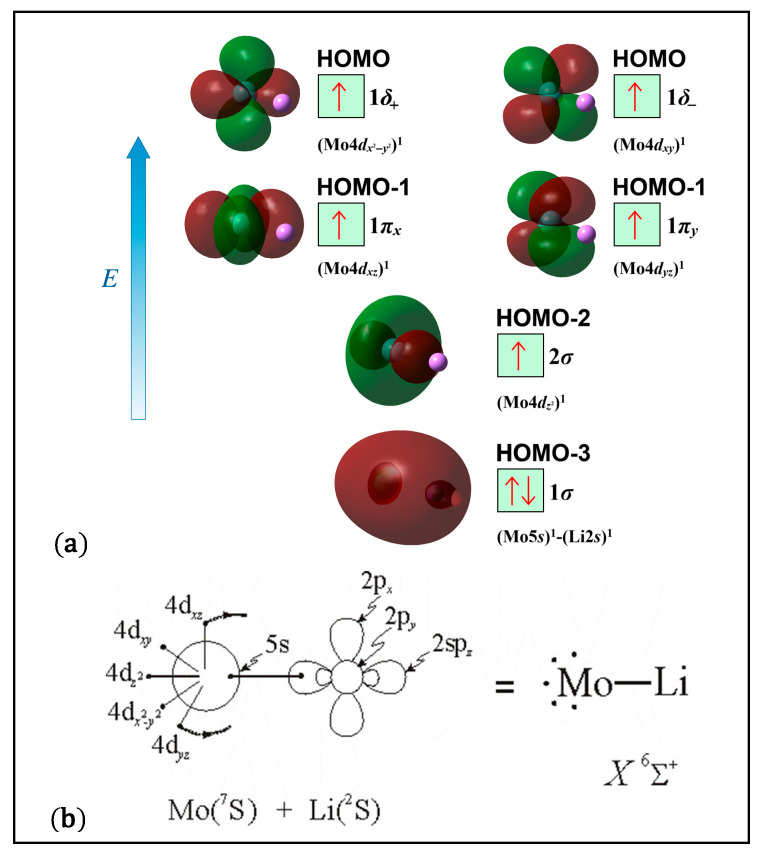
(**a**) MO diagrams and (**b**) valence bond Lewis diagram of the ground state XΣ6+ of MoLi at the CASSCF/ aug-cc-pV5Z(-PP) level.

The composition of the molecular orbitals that produce the configuration of the ground state are given in Table 3. A visual representation of these molecular orbitals is given in Scheme 1a, while the corresponding valence bond Lewis (vbL) diagram that summarizes the bonding of this state is given in Scheme 1b. This vbL diagram is based on the leading CSF, the atomic Mulliken distributions, and the molecular orbitals’ composition. More specifically, a single σ bond is formed from the spin coupling between the 5s electron of Mo and the 2s electron of Li. Note that there is hybridization in both atoms, see 1σ bonding orbital in Table 3, i.e., there is 5s5p_z_ hybridization on Mo and 2s2p_z_ hybridization on Li. Overall, about 0.33 e are transferred from the Li atom to the Mo metal.

The bond length of the X state is 2.708 Å, and the corresponding dissociation energy is 24.1 kcal/mol at the MRCISD+Q/aug-cc-pV5Z(-PP) level of theory, see Table 2. This state has also been calculated via coupled cluster theory, including the correlation of the core electron, i.e., C-RCCSD(T)/aug-cc-wcPV5z(-PP), resulting in a dissociation energy of 24.4 kcal/mol [27] along with the ground states of the MoX molecules, where X = Li − F. Thus, the inclusion of the correlation of the 4s^2^4p^6^ electrons of Mo and 1s^2^ of Li does not affect the present calculated dissociation energy obtained via the correlation of the valence-only electrons.

#### 2.2.2. aΣ+8

This excited state is correlated to the same Mo(aS7) + Li(S2) adiabatic atomic products as the ground one. Note that this state at the SA-CASSCF or CASSCF level of theory is repulsive, while at the MRCISD or MRCISD+Q is bound. The leading equilibrium configuration, followed by the Mulliken atomic distributions (Mo/Li) are: |aΣ8+≅ 0.97 $\left.|{1\sigma }^{1}{2\sigma }^{1}{3\sigma }^{1}{1\delta }_{+}^{1}{1\pi }_{x}^{1}1\pi_{y}^{1}1\delta_{-}^{1}\right\rangle$

$$5s^{0.88}5p_{z}^{0.13}5p_{x}^{0.03}5p_{y}^{0.03}4d_{z^{2}}^{0.97}4d_{x^{2}-y^{2}}^{0.99}4d_{xz}^{0.98}4d_{yz}^{0.98}4d_{xy}^{1.00}/2s^{0.91}2p_{z}^{0.08}2p_{x}^{0.01}2p_{y}^{0.01}$$

**Scheme 2:**
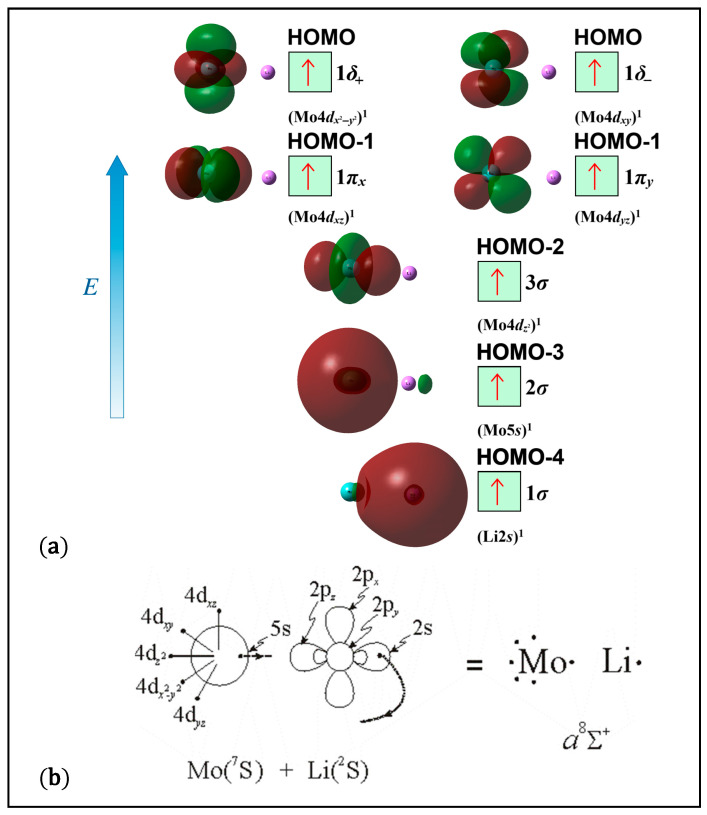
(**a**) MO diagrams and (**b**) valence bond Lewis diagram of the αΣ+8 state of MoLi at the CASSCF/aug-cc-pV5Z(-PP) level.

The composition of the molecular orbitals that produce the configuration of the excited state are given in Table 3, and their visual representation is presented in Scheme 2 along with the corresponding vbL diagram, where it is found that the atoms are in their ground atomic states. Notably, not a single covalent or electrovalent bond is formed between the two atoms, for this high spin state to arise, and the two atoms are most likely pulled towards each other by some weak van der Waals interactions. This interaction is depicted in the 1σ^1^ and 2σ^1^ orbitals. Specifically, the lowest-energy molecular orbital (HOMO-4 = 1σ) orbital has a composition that clearly gives it the character of the Li atom’s 2s orbital, but small electron density is transfer toward the Mo atom via its 5p_z_ empty orbitals, see Table 3. Similarly, the 2σ orbital, which corresponds to the 5s of Mo, interacts with the empty 2p_z_ orbital of Li. This renders the Li atom a center of electron density accumulation (low potential); hence, a very small electronic charge is transferred onto it, i.e., 0.03 e. The resulting interaction has a bond length of 3.354 Å, and a dissociation energy of merely 2.26 kcal/mol at the MRCISD + Q/aug-cc-pV5Z(-PP) level of theory, see also Table 2.

#### 2.2.3. AΣ+6 and CΣ+6

The second excited state, AΣ+6, of MoLi correlates to the Mo(aS5) + Li(S2) adiabatic atomic products and retains these atomic states in the equilibrium. The state is multireference since its leading equilibrium CASSCF configuration has a coefficient of 0.72, i.e., |AΣ6+≅ 0.72 $\left.|{1\bar{\sigma }}^{1}{2\sigma }^{1}{3\sigma }^{1}{1\delta }_{+}^{1}{1\pi }_{x}^{1}1\pi_{y}^{1}1\delta_{-}^{1}\right\rangle$, while the CASSCF Mulliken atomic distributions (Mo/Li) are:

$$5s^{0.98}5p_{z}^{0.17}5p_{x}^{0.03}5p_{y}^{0.03}4d_{z^{2}}^{0.94}4d_{x^{2}-y^{2}}^{0.98}4d_{xz}^{0.96}4d_{yz}^{0.96}4d_{xy}^{0.98}/2s^{0.59}2p_{z}^{0.28}2p_{x}^{0.02}2p_{y}^{0.02}$$

**Scheme 3:**
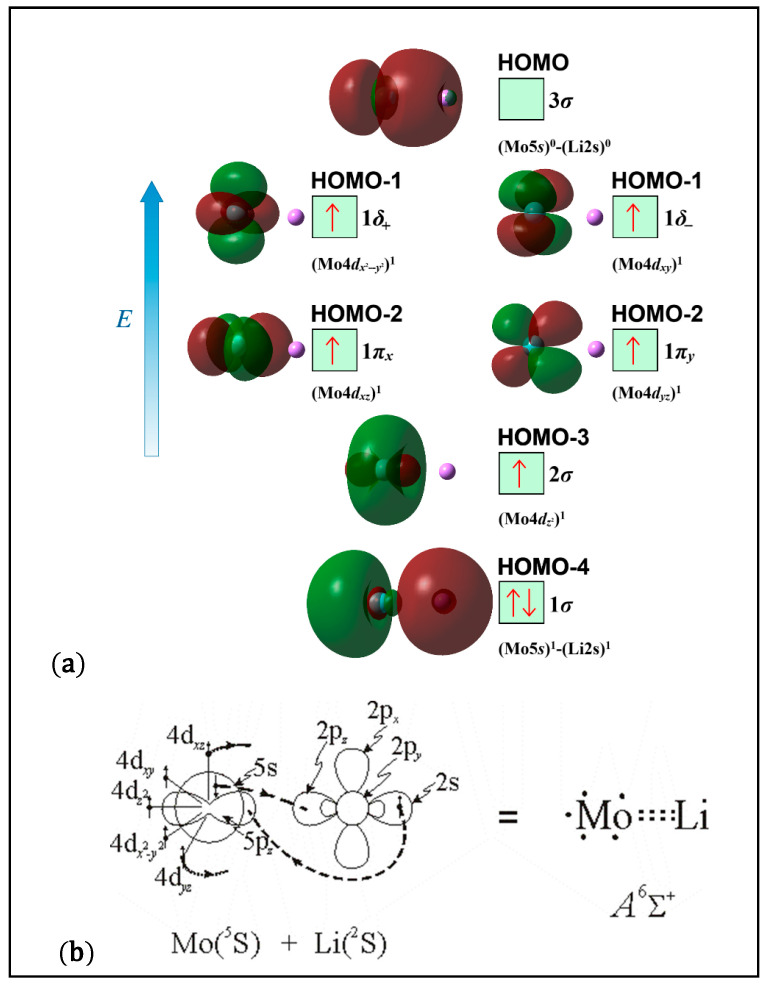
(**a**) MO diagrams and (**b**) valence bond Lewis diagram of the AΣ6+ state of MoLi at the CASSCF/aug-cc-pV5Z(-PP) level.

The bonding in this state is summarized in the vbL diagram of Scheme 3, based on the leading CSF, the atomic Mulliken distributions, and the molecular orbitals’ composition, see Table 3, and their visualization in Scheme 3. There is 5s4d_0_5p_z_ hybridization on Mo and 2s2p_z_ hybridization on Li, as can be seen in the 1σ, 2σ, and 3σ molecular orbitals. In the 1σ molecular orbital, an electron charge is transferred from (**5s**5p_z_)^1^ of Mo to (2s**2p_z_**)^0^ of Li, while in the 3σ molecular orbital, an electron charge is transferred back from (**2s**2p_z_)^1^ of Li to (5s4d_0_**5p_z_**)^0^ of Mo. Thus, we can regard that two half dative σ bonds are formed from the dislocation of Mo’s 5s electron density inside the vacant 2$p_{z}$ orbital of Li and from the Li’s 2s electron density inside the vacant 5$p_{z}$ orbital of Mo resulting in a total charge transfer of about 0.07 e from the Li atom to the Mo metal. Overall, we can regard that the molecule has a bond order of 1, a bond length of 3.027 Å, and a dissociation energy of 19.04 kcal/mol at the MRCISD+Q method.

The CΣ+6 excited state correlates to the Mo(aD5;5s24d4) + Li(S2) adiabatic atomic products. The leading equilibrium CASSCF configuration is: |CΣ6+≅ 0.77 $\left.|{1\sigma }^{2}{2\sigma }^{1}{1\delta }_{+}^{1}{1\pi }_{x}^{1}1\pi_{y}^{1}1\delta_{-}^{1}\right\rangle$. It is lying 47.7 kcal/mol and 12.9 kcal/mol above the two lowest in energy states with the same symmetry, i.e., XΣ+6(1) and AΣ+62, respectively. In this state, a σ^2^ dative bond is formed from the 5s^2^ (Mo) → 2p_z_ (Li), and a σ^1^ half dative bond is formed from the 2s^1^ (Li) → 5p_z_^0^ (Mo), see MO in Table 3. Thus, the bond order is 1.5, while there is a strong hybridization, i.e., 2s2p_z_ in Li and 5s4d_0_5p_z_ in Mo. The MRCISD+Q bond length of the CΣ+6 state is 3.029 Å, i.e., almost the same as the bond distance of the AΣ+62 state, but the dissociation energy of the CΣ+6 state is 13.24 kcal/mol, i.e., it is smaller by about 6 kcal/mol than the corresponding value of the AΣ+6 state.

#### 2.2.4. bΠ8

The state correlates to the Mo(aS7) + Li(P2, ±1) adiabatic atomic products and retains this character in the whole calculated PEC. The leading equilibrium CASSCF configuration of the bΠ8 state and the Mulliken atomic distributions (Mo/Li) are:

Π8≅121σ12σ11δ+11πx12πx11πy11δ−1+1σ12σ11δ+11πx11πy12πy11δ−1

$$5s^{0.72}5p_{z}^{0.05}5p_{x}^{0.56}5p_{y}^{0.01}4d_{z^{2}}^{0.93}4d_{x^{2}-y^{2}}^{0.99}4d_{\mathrm{xz}}^{0.97}4d_{\mathrm{yz}}^{0.96}4d_{\mathrm{xy}}^{0.99}/2s^{0.20}2p_{z}^{0.07}2p_{x}^{0.42}2p_{y}^{0.02}\left(B_{1}\mathrm{symmetry}\right)$$

$$5s^{0.72}5p_{z}^{0.05}5p_{x}^{0.01}5p_{y}^{0.56}4d_{z^{2}}^{0.93}4d_{x^{2}-y^{2}}^{0.99}4d_{\mathrm{xz}}^{0.96}4d_{\mathrm{yz}}^{0.95}4d_{\mathrm{xy}}^{0.99}/2s^{0.20}2p_{z}^{0.07}2p_{x}^{0.02}2p_{y}^{0.42}\left(B_{2}\mathrm{symmetry}\right)$$

**Scheme 4:**
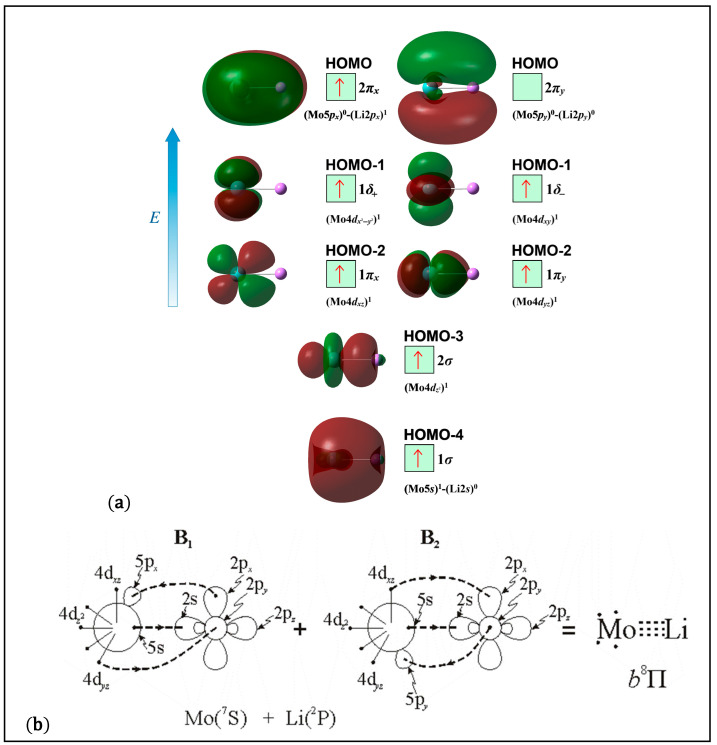
(**a**) MO energy diagrams and (**b**) valence bond Lewis diagram of the bΠ8 state of MoLi at the CASSCF/aug-cc-pV5Z(-PP) level.

The composition of the molecular orbitals that produce the configuration of the excited state are given in Table 3, where an intense 2s**2p_z_** hybridization is observed on Li. Additionally, there is also a 5s4d_0_**5p_z_** hybridization of Mo, see Table 3 and Scheme 4. The bonding is summarized in the vbL diagram of Scheme 4. Specifically, a σ^1^ dative half bond is formed via the donation of electron density from the lone 5s electron of Mo into an empty 2sp_z_ hybrid orbital of Li. In addition, two π^1^ dative half bonds are also formed, either through the donation of electron density from the lone 2p_x_^1^ electron of Li into an empty 5p_x_ orbital of Mo and the electron donation from 4d_yz_^1^ (Mo) to 2p_y_^0^ (Li) corresponding to B_1_ symmetry or 2p_y_^1^ (Li) → 5p_y_^0^ and 4d_xz_^1^ (Mo) → 2p_x_^0^ (Li). Thus, about 0.3 e is transferred to 5p_x_ and 5p_y_, see the Mulliken analysis above. Overall, 0.23 e is transferred from Li to Mo, and the formed bonding order is 1.5, i.e., three half covalent bonds. The bond length is 2.654 Å, and the MRCISD+Q dissociation energy is 30.2 kcal/mol. This is very interesting due to the high multiplicity of spin of this state.

#### 2.2.5. cΠ4 and Π4(2)

**Figure 6:**
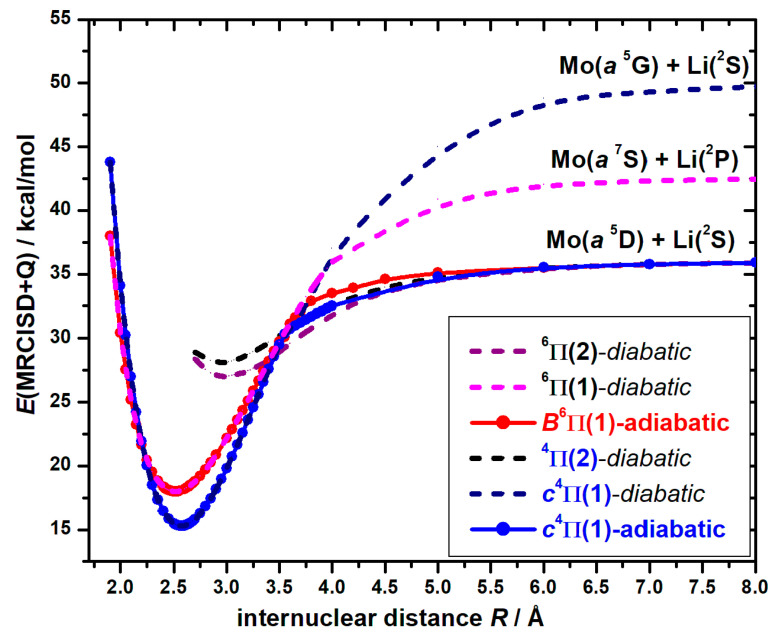
PECs of the adiabatic and diabatic $c^{4}\Pi \;and\;B^{6}\Pi$ states of MoLi at the MRCISD+Q/aug-cc-pV5Z(-PP) computational level. The Mo (a ^7^S) + Li (^2^S) limit, to which the X^6^Σ^+^ state correlates, is used to define the zero of energy for this figure.

The c ^4^Π state correlates to the Mo(aD5;5s24d4) + Li(S2) adiabatic atomic products, however, it does not retain this character in the whole PEC. At about 3.5 Å an avoided crossing is observed with an excited ^4^Π(2) state that correlates to Mo(aG5;5s14d5) + Li(S2), see Figure 2 and Figure 5. In Figure 6, the adiabatic cΠ4(1) and the two diabatic cΠ(1)4 and Π4(2) have been plotted, where a crossing is clearly observed.

At the minimum of the cΠ4 state, its leading CASSCF configuration and Mulliken distributions (Mo/Li) are: |cΠ4≅0.622 |1σ21δ+11πx21πy11δ−1+|1σ21δ+11πx11πy21δ−1=0.622(B1+B2)

$$5s^{1.41}5p_{z}^{0.09}5p_{x}^{0.05}5p_{y}^{0.03}4d_{z^{2}}^{0.60}4d_{x^{2}-y^{2}}^{0.77}4d_{xz}^{1.54}4d_{yz}^{1.15}4d_{xy}^{0.76}/2s^{0.30}2p_{z}^{0.03}2p_{x}^{0.13}2p_{y}^{0.06}\left(B_{1}\mathrm{symmetry}\right)$$

$$5s^{1.41}5p_{z}^{0.09}5p_{x}^{0.03}5p_{y}^{0.05}4d_{z^{2}}^{0.60}4d_{x^{2}-y^{2}}^{0.77}4d_{xz}^{1.15}4d_{yz}^{1.54}4d_{xy}^{0.76}/2s^{0.30}2p_{z}^{0.03}2p_{x}^{0.06}2p_{y}^{0.13}\left(B_{2}\mathrm{symmetry}\right)$$

**Scheme 5:**
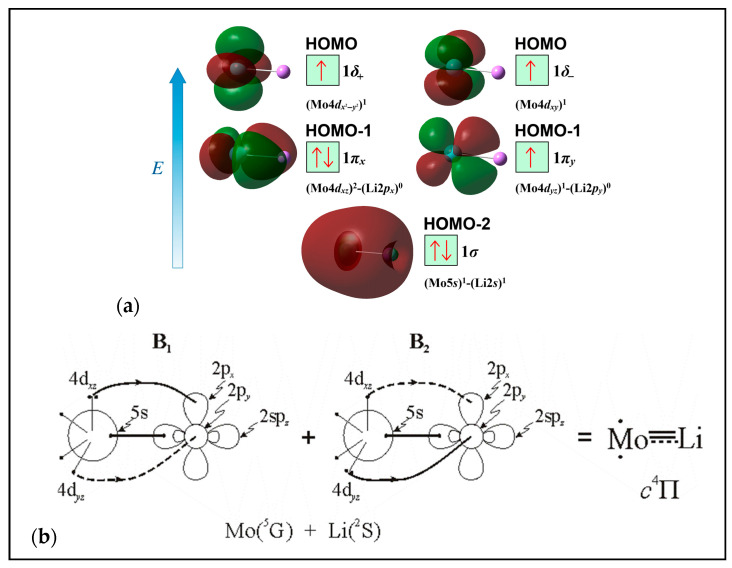
(**a**) MO diagrams and (**b**) valence bond Lewis diagram of the cΠ4(1) state of MoLi at the CASSCF/aug-cc-pV5Z(-PP) level.

The bonding is summarized in Scheme 5, and is formed from the spin coupling between the 5s electron of Mo and the 2s electron of Li, resulting in a σ^2^ covalent bond, where about 0.65 e is transferred via this σ to the Μο atom and two dative bonds, i.e., π^2^ and π^1^. The π bonds are either a full dative π bond from the dislocation of the $4d_{xz}$ electron pair of Mo inside the $2p_{x}$ vacant orbital of Li, and a half dative π bond from the dislocation of the $4d_{yz}$ single electron of Mo inside the $2p_{y}$ vacant orbital of Li, or the reverse, see MOs. Overall, about 0.42e is transferred from the Li atom to the Mo metal. The resulting interaction has an order of 2.5, a bond length of 2.570 Å, and a dissociation energy of 20.7 kcal/mol at the MRCISD+Q/aug-cc-pV5Z(-PP) level of theory with respect to the correlated atomic products. However, the dissociation energy with respect to the diabatic products, i.e., Mo(aG5;5s14d5) + Li(S2), is 34.7 kcal/mol {=20.68 kcal/mol + 14.05 kcal/mol Mo(aG5;5s14d5aD5;5s24d4 [28,29]}. Finally, the bond distance of the Π4(2) state is 2.954 Å and the corresponding diabatic dissociation energy 7.9 kcal/mol, see Table 2.

#### 2.2.6. dΣ+4

The cΣ+4 state correlates to the Mo(aS5) + Li(S2) adiabatic atomic products and retains this character in its equilibrium. The leading equilibrium CASSCF configuration and the corresponding Mulliken atomic distributions (Mo/Li) are |cΣ4+⟩≅ 0.70$|{1\sigma }^{2}{2\sigma }^{1}{1\delta }_{+}^{1}{1\bar{\pi }}_{x}^{1}1\pi_{y}^{1}1\delta_{-}^{1}\rangle$,

$$5s^{1.32}5p_{z}^{0.09}5p_{x}^{0.02}5p_{y}^{0.02}4d_{z^{2}}^{1.02}4d_{x^{2}-y^{2}}^{1.00}4d_{xz}^{0.92}4d_{yz}^{0.92}4d_{xy}^{1.00}/2s^{0.48}2p_{z}^{0.05}2p_{x}^{0.07}2p_{y}^{0.07}$$

**Scheme 6:**
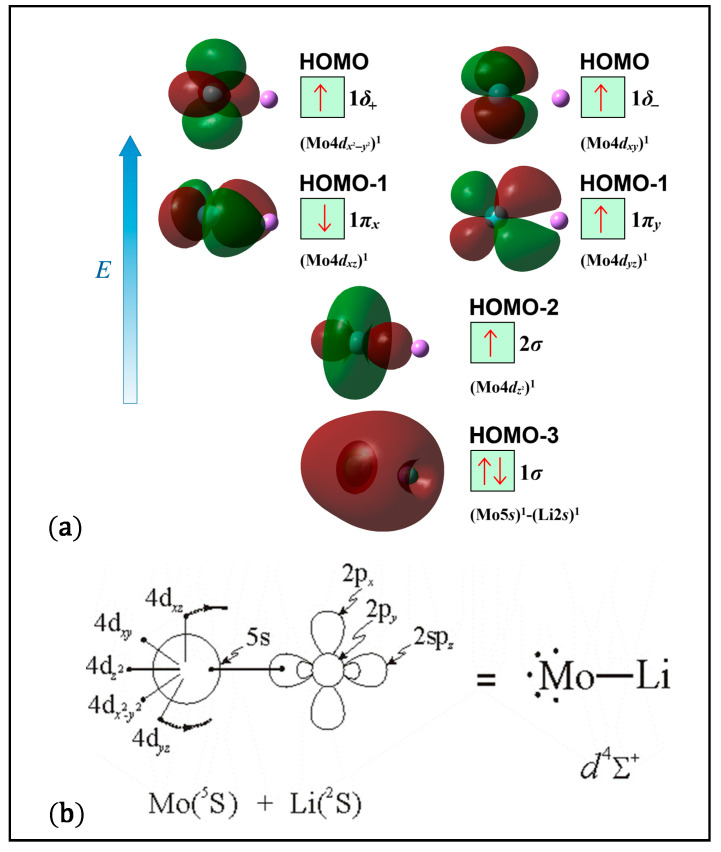
(**a**) MO diagrams and (**b**) valence bond Lewis diagram of the dΣ+4 state of MoLi at the CASSCF/aug-cc-pV5Z(-PP) level.

Considering the composition of the molecular orbitals (see Table 3) and the population analysis, the bonding is formed as it is depicted in Scheme 6. A single σ bond is formed between the 5s electron of Mo and the 2s electron of Li as in the case of the ground state and a total of about 0.36e are transferred from the Li atom to the Mo metal. The bond length of the cΣ+4 state is 2.674 Å, and the corresponding dissociation energy is 11.9 kcal/mol at the MRCISD+Q/aug-cc-pV5Z(-PP) level of theory. Its dipole moment is 3.27 D, a relatively high value.

Comparing the ground state XΣ+6 and the cΣ+4 state, both present a similar σ bond with bond lengths of 2.708 Å and 2.674 Å, respectively, however, the strength of the resulting bond of the c state appears to be half of that of the X state, even though the X states have an σ bond length elongated by 0.03 Å. The two states differ in hybridization. Specifically, the $4d_{z^{2}}$ orbital of Mo participates to an extent in the bonding of the c state, while this does not happen in the X state, see molecular orbitals in Table 3; thus, this hybridization extends the space of the molecular orbitals and may weaken the binding energy of the formed bond.

#### 2.2.7. BΠ6 and Π6(2)

The BΠ6 state of MoLi correlates to the Mo(aD5) + Li(P2) adiabatic atomic products. Around 3.5 Å, there is an avoided crossing with an excited Π62, and its character changes to Mo(aS7) + Li(P2) atomic products, in which the Mo atom is in ground state and the Li atom is in its first excited one. In Figure 6, the adiabatic BΠ6(1) and the two diabatic BΠ(1)6 and Π6(2) have been plotted where the crossing is clearly observed. The leading equilibrium CASSCF configuration of the BΠ(1)6 state, followed by the Mulliken atomic distributions (Mo/Li), are:

|BΠ6⟩≅0.732|1σ12σ11δ+11πx21πy11δ−1+|1σ12σ11δ+11πx11πy21δ−1=0.732(B1+B2)

$$5s^{0.81}5p_{z}^{0.07}5p_{x}^{0.16}5p_{y}^{0.01}4d_{z^{2}}^{0.95}4d_{x^{2}-y^{2}}^{0.99}4d_{xz}^{1.23}4d_{yz}^{0.96}4d_{xy}^{0.99}/2s^{0.15}2p_{z}^{0.05}2p_{x}^{0.54}2p_{y}^{0.03}\left(B_{1}\mathrm{symmetry}\right)$$

$$5s^{0.81}5p_{z}^{0.07}5p_{x}^{0.01}5p_{y}^{0.16}4d_{z^{2}}^{0.95}4d_{x^{2}-y^{2}}^{0.99}4d_{xz}^{0.96}4d_{yz}^{1.23}4d_{xy}^{0.99}/2s^{0.15}2p_{z}^{0.05}2p_{x}^{0.03}2p_{y}^{0.54}\left(B_{2}\mathrm{symmetry}\right)$$

**Scheme 7:**
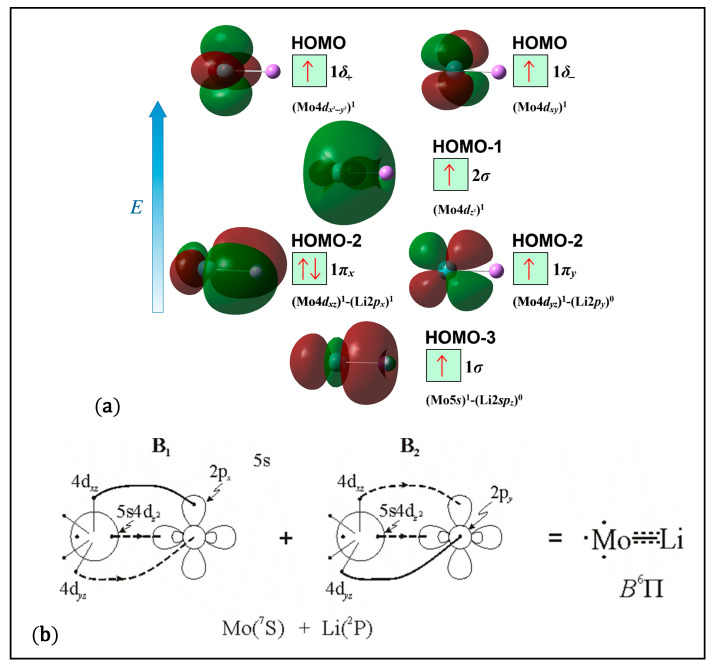
(**a**) MO diagrams and (**b**) valence bond Lewis diagram of the BΠ6 state of MoLi at the CASSCF/aug-cc-pV5Z(-PP) level.

The composition of the valence molecular orbitals and their visualization are given in Table 3 and plotted in Scheme 7 along with the vbL diagram. A half dative σ bond is formed from the (**5s**$4d_{z^{2}}$)^1^ hybridized orbital of Mo to the vacant **2s**$p_{z}$ hybrid orbital of Li, and either a π covalent bond from the spin coupling between a $4d_{xz}$ electron of Mo and a $2p_{x}$ electron of Li and a half dative π bond from the dislocation of the $4d_{yz}$ single electron of Mo in the vacant $2p_{y}$ orbital of Li or the reverse. A total of about 0.20 e is transferred from the Li atom to the Mo metal. Thus, the bond order is 2, resulting in a bond length of 2.513 Å and an adiabatic (diabatic) dissociation energy of 18.0(24.7) kcal/mol at the MRCISD+Q/aug-cc-pV5Z(-PP) level of theory. Finally, the bond distance of the Π6(2) state is about 0.5 Å elongated compared to the BΠ(1)6 state, i.e., at 2.981 Å, and the corresponding diabatic dissociation energy is 9.0 kcal/mol.

#### 2.2.8. Σ+2 and Π2

The Σ+2 and Π2 states correlate to the Mo(aD3 or aG3) + Li(S2) adiabatic atomic products and retain this character in equilibrium since no avoided crossings are observed for either state. It should be noted that the atomic states of Mo ^3^D and ^3^G are practically degenerate; their lowest J term differs only 0.05 kcal/mol, i.e., ^3^D_1_ is lower than ^3^G_3_, while with respect to their average j term, ^3^G is lower than ^3^D by 0.23 kcal/mol [28,29]. Since the spin-orbit coupling has not been included, our results correspond to the Mj average term, and thus, we may consider a^3^G as the lowest one even though a^3^D and a^3^G are energetically degenerate.

For the Σ+2(1) state, its leading equilibrium CASSCF configurations and the Mulliken atomic distributions (Mo/Li) are: |dΣ2+⟩≅ 0.51 $\left|{1\sigma }^{2}{2\sigma }^{1}{1\delta }_{+}^{1}{1\bar{\pi }}_{x}^{1}1{\bar{\pi }}_{y}^{1}1\delta_{-}^{1}\right\rangle -0.32\left|{1\sigma }^{2}{2\sigma }^{1}{1\delta }_{+}^{1}{1\bar{\pi }}_{x}^{1}1\pi_{y}^{1}1{\bar{\delta }}_{-}^{1}\right\rangle$

$$5s^{1.43}5p_{z}^{0.08}5p_{x}^{0.03}5p_{y}^{0.03}4d_{z^{2}}^{1.07}4d_{x^{2}-y^{2}}^{086}4d_{xz}^{1.00}4d_{yz}^{1.00}4d_{xy}^{0.86}/2s^{0.40}2p_{z}^{0.05}2p_{x}^{0.06}2p_{y}^{0.06}$$

**Scheme 8:**
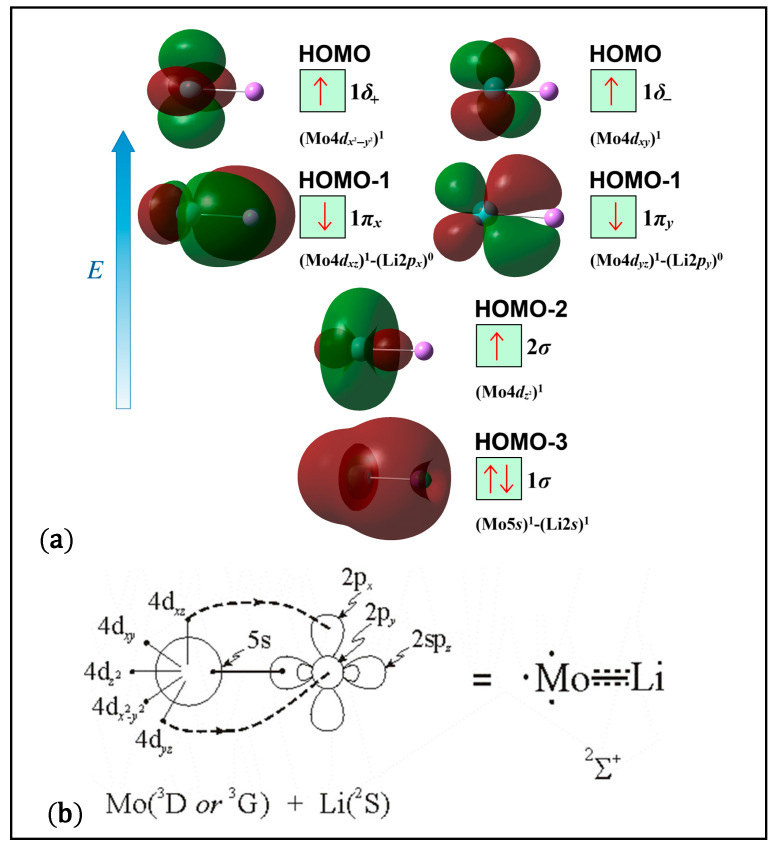
(**a**) MO diagrams and (**b**) valence bond Lewis diagram of the Σ+2 state of MoLi at the CASSCF/aug-cc-pV5Z(-PP) level.

The state is a very multireference state, i.e., c_0_ = 0.51. The bonding is summarized in the following vbL diagram of Scheme 8. In short, a σ^2^ covalent bond is formed, i.e., 5s^1^(Mo)- 2s^1^(Li), as well as two half π dative bonds, $4d_{xz}$^1^ → 2p_x_^0^ and $4d_{yz}$^1^ → 2p_y_^0^, while about 0.39 e is transferred from the Li atom to the Mo atom. The bond order is 2, while the bond length and the dissociation energy are 2.635 Å, and 31.4 kcal/mol, respectively, at the MRCISD+Q/aug-cc-pV5Z(-PP) level of theory.

The Π2(1) state is a highly multireference state, as it is shown by the leading equilibrium CASSCF configuration |dΠ2⟩≅0.502{|1σ21δ+11πx21π-y11δ−1+|1σ21δ+11π¯x11πy21δ−1|⟩ = 0.502(B1+B2). The Mulliken atomic distributions are:

$$5s^{1.39}5p_{z}^{0.09}5p_{x}^{0.03}5p_{y}^{0.03}4d_{z^{2}}^{0.59}4d_{x^{2}-y^{2}}^{0.83}4d_{xz}^{1.39}4d_{yz}^{1.18}4d_{xy}^{0.83}/2s^{0.35}2p_{z}^{0.04}2p_{x}^{0.08}2p_{y}^{0.09}\;(B_{1}\;\mathrm{symmetry})$$

$$5s^{1.39}5p_{z}^{0.09}5p_{x}^{0.03}5p_{y}^{0.03}4d_{z^{2}}^{0.59}4d_{x^{2}-y^{2}}^{0.83}4d_{xz}^{1.18}4d_{yz}^{1.39}4d_{xy}^{0.83}/2s^{0.35}2p_{z}^{0.04}2p_{x}^{0.09}2p_{y}^{0.08}\;(B_{2}\;\mathrm{symmetry})$$

**Scheme 9:**
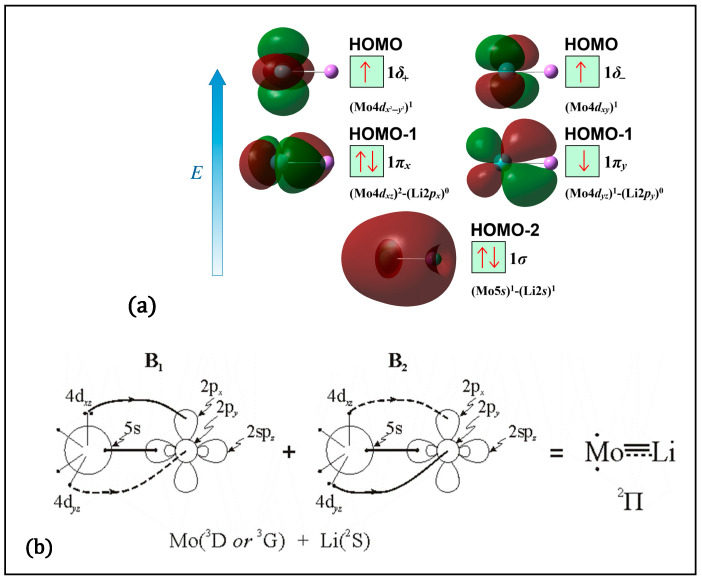
(**a**) MO diagrams and (**b**) valence bond Lewis diagram of the Π2 state of MoLi at the CASSCF/aug-cc-pV5Z(-PP) level.

The bonding consists of a σ^2^ covalent bond, i.e., 5s^1^(Mo)- 2s^1^(Li), a dative π^2^ bond, and a dative π^1^ bond, i.e., $4d_{xz}$^2^ → 2p_x_^0^ and $4d_{yz}$^1^ → 2p_y_^0^ or $4d_{yz}$^2^ → 2p_y_^0^ and $4d_{xz}$^1^ → 2p_x_^0^, while about 0.40 e are transferred from the Li atom to the Mo atom, see Scheme 9. The resulting bond has an order of 2.5, a length of 2.589 Å, and a dissociation energy of 27.0 kcal/mol at the MRCISD+Q level of theory.

#### 2.2.9. Trends and Electronic Spectra

**Figure 7:**
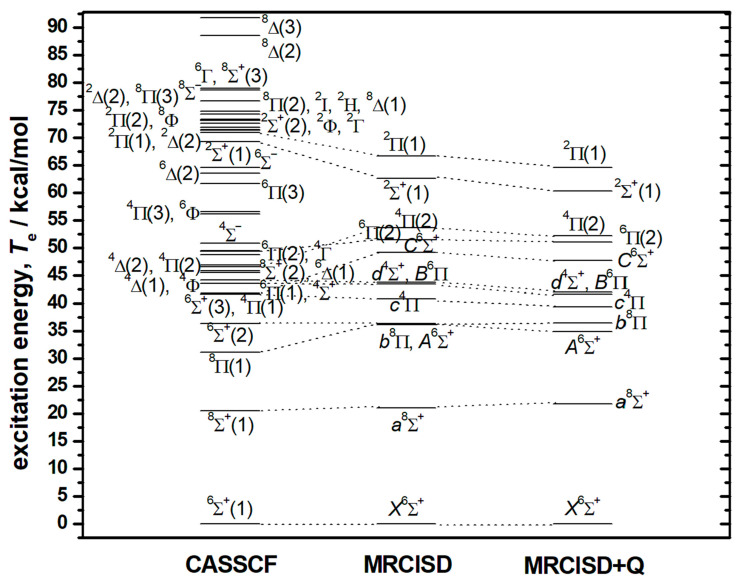
Relative energy levels (diabatic excitation energies) of all computed states of MoLi with respect to its lowest-lying state at various levels of computation.

The adiabatic excitation energies, i.e., relative energy levels of the 41 calculated electronic states at the CASSCF, MRCISD, and MRCISD+Q levels are depicted in Figure 7. It is obvious that there are regions around 45 kcal/mol (=2.0 eV) and 75 kcal/mol (=3.3 eV) where the electronic spectrum is very intense. The five lowest in energy states are clearly separated for the remaining excited states in all three methods. Our best methodology is the MRCISD+Q method, and the states are named according to the MRCISD+Q order. Some states present avoided crossing at around 3.5 Å, for instance, cΠ4 and BΠ6 states with the Π4(2) and Π6(2) states, see Table 4; ^4^Σ^+^, ^4^Δ(1), and ^8^Δ(2) with the ^4^Σ^+^(2), ^4^Δ(2), and ^8^Δ(3) states, respectively, ^6^Φ and ^6^Δ(2) with higher energy states.

In most cases, the alkali metal atom behaves, as should be expected, as the electropositive participating element. But in the case of the aΣ+8 state of MoLi, which is a high-spin van der Waals state, a negligible excess amount of electron density is accumulated on the alkali metal atom.

**Figure 8:**
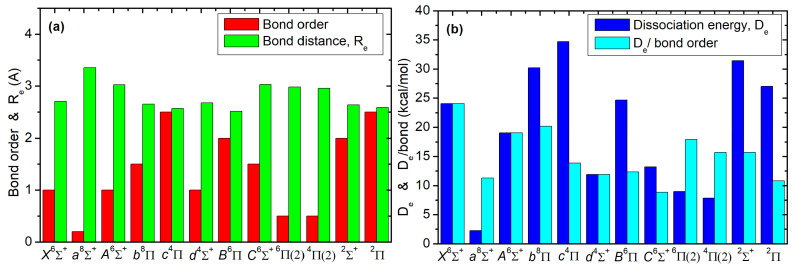
(**a**) Bond order magnitude and bond distances, (**b**) diabatic dissociation energies and dissociation energies/bond of 12 calculated states of MoLi at the MRCISD+Q/aug-cc-pV5Z(-PP) level.

Overall, the correlation between bond distances, bond order, dissociation energies, and specifically how the bonding affects them are depicted in Figure 8. While the Li atom possesses only one electron, it can form a variety of bonds, i.e., van der Walls, covalent, and dative. In the MoLi molecule, it was found that it forms from a weak van der Waals bond (aΣ8+) up to 2.5 bonds (cΠ4 and Π2), see Figure 8. In most cases, 2s2p_z_ hybridization on Li and 5s4d_0_5p_z_ or 5s5p_z_ hybridization on Mo are found, while an electron charge is transferred from Li to Mo up to 0.4 e. The bond distances of the calculated states range from 2.513 Å in BΠ6(1) as far as 3.354 Å in aΣ8+(1), see Table 2 and Figure 8. Generally, it is observed that the excited Li(^2^P) atom forms the shortest bonds because its empty 2s^0^ orbital can easily accept an electron charge from the 5s4d_0_5p_z_ orbitals of Mo, and thus, a strong σ dative bond can be formed. Finally, the adiabatic dissociation energies and diabatic ones in the cases of cΠ4 and BΠ6 are depicted in Figure 8b. The calculated dissociation energies range from 2.26 kcal/mol to 34.7 kcal/mol, see Figure 8. The dissociation energy per bond is obtained if the diabatic dissociation energy is divided with the bond order, and the lowest energy state (XΣ6+, AΣ6+, and bΠ8) is, on average, 21 kcal/mol, while the remaining calculated states are, on average, 13 kcal/mol. Finally, the van der Waals interaction is calculated at 2.3 kcal/mol. All these values can be used to evaluate the interaction energy of the chemical or adsorbed Mo-Li bonds in materials and complexes.

#### 2.2.10. Comparison of MoLi with CrLi and MoB Diatomic Molecules

As mentioned in the beginning, there are no previous experimental or theoretical studies on the species of the diatomic MoLi molecule. Moreover, as far as we know, no previous studies on MoNa and MoK exist in the literature. Below we compare our results on MoLi with the corresponding data of the isovalent CrLi and the MoB molecules. The second one contains the B atom, which has one unpaired electron as the Li atom does.

As expected, the results obtained for MoLi bear impressive resemblance to those regarding its isovalent brethren CrLi. Namely, the electronic spectrum of this molecule, also, consists of a X^6^Σ^+^ ground term, whilst its first excited state is of a^8^Σ^+^ symmetry [30]. The reported bond distances, dissociation energies, and dipole moments for the aforementioned two states are R_e_ = 4.87(1) a_0_ ≈ 2.58 Å, D_e_ = 8769(140) cm^−1^ ≈ 1.09 eV, and μ = 3.3(2) D, with a Li^δ+^Cr^δ−^ dipolar character for the ground state X^6^Σ^+^ of CrLi at the CCSDTQ/ aug-cc-pVQZ level of theory and R_e_ = 6.50(2) a_0_ ≈ 3.44 Å, D_e_ = 553(11) cm^−1^ ≈ 0.07 eV, and μ = 0.67(3) D for the first excited state a^8^Σ^+^ at the MRCISD(+Q)/aug-cc-pVQZ level of theory [30]. Additionally, the same ground state, CrLi X^6^Σ^+^, has been reported to correspond to an R_e_ = 2.751 Å and a D_e_ = 0.683 eV at the MRCI level, while a reported ^6^Π state, approximately 1.56 eV above the ground term, corresponds to an R_e_ = 3.132 Å and a D_e_ = 0.201 eV at the MRCI level of theory in a different examination [31]. The bonding schemes of these states of CrLi appear to be identical to the corresponding ones of MoLi; |XΣ6+⟩~|1σ22σ11δ+11πx11πy11δ−1⟩, and |aΣ8+⟩~|1σ12σ13σ11δ+11πx11πy11δ−1⟩ [30,31], and |BΠ6⟩~{|1σ12σ11δ+11πx21πy11δ−1|+|1σ12σ11δ+11πx11πy21δ−1⟩} [31].

It is interesting that for a homologous species of an electropositive element interacting with Mo, i.e., MoB, the ground state was found to be that of a X^6^Π term, with quite a shorter R_e_ = 1.968 Å, a D_e_ = 2.18 eV, and a μ = 2.7 D, while its dominant configuration, i.e., (80%)1σ^2^2σ^1^3σ^1^1δ^2^1π^3^, attributes a bonding scheme to the molecule MoB, which is similar to that of the B^6^Π excited state of MoLi, namely that of a σ half-bond (from one of Mo’s σ symmetry electron density inside one of B’s σ symmetry vacant orbitals), and lateral π interactions (1σ = B2s(σ), 2σ = Mo4d(σ) + B(2s(σ)+2p(σ)), 3σ = Mo4s(σ), 1δ = B4d(δ), 1π = Mo4d(π) + B2p(π), MPA: 5s^0^.^81^4d^4^.^86^/2s^1^.^54^2p^1^.^54^), at the CASPT2+DKH/4ζ−8s7p5d3f2g-ANO-RCC_Mo_/4ζ−5s4p3d2f_B_ level [32]. Similar results were obtained in a previous work of ours [22] for MoB, i.e., ground state is of X^6^Π symmetry, has a bond length *R*_e_ = 1.973 Å, a dissociation energy *D*_e_ = 2.247 eV, and a dipole moment *μ* = 2.221 D, at the B3LYP/aug-cc-pVQZ(-PP) level of theory, as well as a recent study of the molecule, [27] where the results obtained bear impressive resemblance to the previous ones, i.e., *R*_e_ = 1.959 Å, *D*_e_ = 2.05 {2.09} eV, and *μ* = 2.31 {2.47} D, using the C-RCCSD(T) {C-MRCISD+Q} methodologies, with an identical bonding scheme comprising one σ half-bond, one π covalent bond, and one π half-bond [27].

#### 2.2.11. Mo-Li Bonds in Complexes and Solid State

The Mo–Li bond distances depend on the specific compound and the coordination environment. Furthermore, in many inorganic structures, the Mo–Li interaction is not a direct bonding pair, even though electronic interactions take place between the metal atoms in every instance. To name a few, in organometallic or in cluster compounds, Mo and Li are directly bonded or interacting through bridging ligands. Typical Mo–Li bond distances observed in organometallic complexes, generally range from 2.6 Å to 2.9 Å, depending on the specific ligands and structural configurations [33,34,35]. For instance, in the case of the complex unit Mo_2_LiHC, the Mo–Li bond distances have been determined through experimental and computational studies, and their averaged values span a range from 2.856(7) Å to 3.03(5) Å, owing to (a) σ(Mo–Mo) → s(Li), (b) π(Mo–Mo) → s(Li), (c) δ(Mo–Mo) → p_z_(Li), (d) σ(Mo–H) → s(Li), and (e) σ(Mo–C) → s(Li) natural orbital donor/acceptor electronic interactions [33]. Moreover, in a similar study of the complex Mo_2_LiH_2_, Mo–Li bond distances have also been determined experimentally and computationally. The experimental Mo–Li bond lengths range from 2.91(2) Å to 3.24 Å, while computational models predict approximately Mo-Li = 2.97–3.25 Å, depending on the coordination environment, as a result from (a) σ(Mo–Mo) → p_y_(Li), (b) π_yz_(Mo–Mo) → p_y_ (Li), (c) δ(Mo–Mo) → p_x_(Li), and (d) σ(Mo–H) → p_z_(Li) natural orbital donor/acceptor interactions [34]. Note also that in these homologous cluster compounds, there is hydride, carbanide, or carbonyl bridging and not a direct bonding of Mo-Li (their interactions are purely a result of electron delocalization) [33,34]. Additionally, when direct bonding explicitly between Mo-Li has been reported, the bond length Mo1–Li1 was 2.640(8) Å, and the effective bond order (eBO) of Mo–Li–Mo is 0.57 [35]. The Mo-Li bond distances calculated here are within the Mo–Li bond distances observed in organometallic complexes and can provide information on the type of Mo-Li bonding. Additionally, regarding the dissociation energy of the Mo–Li bond, where there are no reports, the present study can evaluate the dissociation energy of the Mo-Li bonds within organometallic compounds, i.e., the data obtained via the present accurate study of the diatomic molecule can be correlated with the corresponding MoLi complexes, providing, in this way, valuable information for the elucidation of the nature of the interactions taking place in such structures, through the assessment of structural characteristics and energetics.

Understanding the interactions between lithium and molybdenum in solid-state systems and diffuse Li in the material can provide insights into their potential applications in energy storage and catalysis [36,37,38,39]. Specifically, the adsorption of Li on MoS_2_ [36] in MoS_2(1-x)_Se_2x_ alloys [37] has been studied, and it was found that Li loses the 2s^1^ electron, [36,37], such as in the present calculations, where in some states, the 2s orbital of Li includes only 0.15e. Moreover, depending on the type of material and the defect, it was found that the adsorption energy in defect monolayer MoS_2_, when Li substitutes a S atom and interacts with three Mo atoms, is calculated in the range of 2.81–3.80 eV [36], and per Mo-Li moiety is in the range 22–29 kcal/mol, which is the range of the present calculated dissociation energies. Furthermore, in first-principle characterization of absorption of Li on Mo(110) surfaces, bond lengths d_Li–Mo_, i.e., the distance between Li and the closest Mo atom, have been reported to range between 2.547 Å and 3.151 Å [40], and in the characterization of the liquid Li-solid Mo(110) interface, Li-Mo distances ranged from 2.229 Å, up to 2.663 Å [41]. Consequently, this study can serve as a valuable guide for understanding such interactions. Overall, the present study can add physical insight into Mo-Li bonding, and it can enhance our understanding of these interactions in materials, offering valuable information for possible uses in lithium-based energy storage and catalytic material design.

## 3. Computational Details and Methodology

The electronic structure of 41 electronic states of the MoLi molecule were investigated using the augmented correlation consistent basis sets of quintuple-ζ quality: Mo: aug-cc-pV5Z-PP [42], contracted to [8s8p7d5f4g3h2i], and Li: aug-cc-pV5Z [43], contracted to [7s6p5d4f3g2h]. For the Mo atom, the accurate core relativistic pseudo-potentials were used for the 1s^2^2s^2^2p^6^3s^2^3p^6^ electrons, while the 4s^2^4p^6^(5s4d)^6^ electrons of Mo were treated via ab initio calculations. Regarding the inclusion of the relativistic effects, i.e., mass–velocity and Darwin terms, on calculations, we found that for the MoC [23], their consideration via the second order Douglas–Kroll–Hess (DKH2) resulted in the same bond distances and dissociation energies with the data obtained using accurate core relativistic pseudo-potentials of the aug-cc-pV5Z-PP basis set. Thus, we expect that for MoLi, they will also have no effect on the calculated data. Finally, regarding the inclusion of the spin-orbit coupling for the lowest states, it is found that the ground and the first excited states are the XΣ+6 and aΣ+8 states, which differ a lot, i.e., 21 kcal/mol, so the inclusion of the spin-coupling in their calculation will not affect their relative energy ordering.

At first, 41 states were calculated via state average complete active space self-consistent field (SA-CASSCF) calculations employing the aug-cc-pV5Z(-PP) basis set. The SA-CASSCF reference wavefunctions are built by distributing seven [Mo (4$d^{5}$5$s^{1}$) + Li (2$s^{1}$)] active electrons to ten orbital functions, one “5s” and five “4d”s on Mo, plus one “2s” and three “2p”s on Li. Note that, the “5p” orbitals of Mo have not been included in CASSCF as is customary for this atom [23,44] since previous calculations show that their involvement in static correlation is not important, while they increase the number of CSFs of the CASSCF and the subsequent MRCISD calculation significantly [23]. All calculations were done under $C_{2\upsilon }$ symmetry constraints. The CASSCF wavefunctions have the correct axial angular momentum symmetry, i.e., |Λ| = 0 (Σ^+^, Σ^−^), 1 (Π), 2 (Δ), 3 (Φ), 4 (Γ), 5 (H), 6(Ι). So, Σ^+^ and Σ^−^ correspond to A_1_ and A_2_ symmetries, respectively; Δ, Γ, and Ι are linear combinations of A_1_ and A_2_ symmetries; Π, Φ, and H are linear combinations of B_1_ and B_2_ symmetries [45,46]. SA-CASSCF was applied for states within the same multiplicity of spin. Two groups were obtained; the first one contains the A_1_ and A_2_ symmetries, which includes Σ^+^, Σ^−^, Δ, Γ, and Ι, and the second one contains the B_1_ and B_2_ symmetries, which includes Π, Φ, and H.

Then, the lowest in energy states and some selected ones, in total 12 states, were further studied via CASSCF, i.e., without the state average, followed by the multireference configuration interaction plus single and double excitations (CASSCF + single + double replacements = MRCISD) in conjunction with the aug-cc-pV5Z(-PP) basis sets. Generally, MRCISD wavefunctions do not display pure spatial angular momentum symmetry, but their main configuration has the correct spatial angular momentum symmetry. Thus, the corresponding MRCISD wavefunctions have the same axial angular momentum symmetry [47].

Our CASSCF reference spaces are: 3526 (Σ2+), 3460 (Π2), 2272 (Σ4+), 2320 (Π4), 522 (XΣ6+), 492 (Π6), 32 (Σ8+), and 32 (Π8) configuration state functions (CSFs). The corresponding MRCISD spaces are 1 × $10^{8}$ (Σ2+), to 1 × $10^{8}$ (Π2), 9 × $10^{7}$ (Σ4+), 9 × $10^{7}$ (Π4), 3 × $10^{7}$ (XΣ6+), 3 × $10^{7}$ (Π6), 2 × $10^{7}$ (Σ8+), and 3 × $10^{7}$ (Π8) CSFs. The construction of PECs becomes feasible after reducing these numbers by more than one or two orders of magnitude, respectively, via the internal contraction scheme [48]. For simplicity, the notation MRCISD is used for all multireference calculations. Finally, the Davidson correction for unlinked quadruples (+Q) [49,50] was used to ameliorate significant size non-extensivity problems.

The potential energy curves (PECs) of all calculated states are obtained at the CASSCF, MRCISD, and MRCISD+Q levels of theory. Additionally, for some states, avoided crossings were observed, and their diabatic PECs are also plotted. For all methods, bond distances, dissociation energies, and other spectroscopic constants are computed. Dissociation energies are calculated with respect to the adiabatic or diabatic dissociation limit at 15 Å of the PECs. A Dunham analysis [51] is used to extract all spectroscopic constants. The chemical bonding is analyzed. In each case, the bonding has been plotted via 2-D valence bond Lewis (vbL) diagrams and via the 3D plot of the CASSCF valence molecular orbitals using the GaussView 6.1.1 graphical interface [52]. Mulliken population analysis is also included using a small basis set cc-pVTZ(-PP) since this population analysis is basis set dependent, and it provides better data with simple non-augmented basis sets [53]. It is generally agreed that the use of population analyses assists in making comparisons for similar states of the same molecules. The use of the Mulliken analysis is used to compare the charges between different states [53,54]. All calculations were performed by the MOLPRO [55] suite of codes.

## 4. Conclusions and Final Remarks

Employing CASSCF and MRCISD(+Q) methodologies in conjunction with correlation consistent augmented basis sets of quintuple quality, we have explored the electronic structure and bonding of the ground and 40 low-lying states of the MoLi molecule. Bond distances, dissociation energies, dipole moments as well as common spectroscopic constants have been reported while the bonding is analyzed.

The five lowest in energy states are clearly separated for the remaining excited states in all three methods. The ground state is the XΣ+6 state. It has $R_{e}$ = 2.708 Å, $D_{e}$ = 24.1 kcal/mol, $\omega_{e}$ = 316.8 ${\mathrm{cm}}^{-1}$, $\omega_{e}x_{e}$ = 2.11 ${\mathrm{cm}}^{-1}$, and $\mu_{FF}$ = 3.63 D, while a single covalent bond, i.e., 5s^1^(Mo)-2s^1^(Li), is formed.

The dissociation energies of the calculated states range from 2.3 kcal/mol in aΣ8+(1), which is a van der Waals state, to 31.5 kcal/mol in Σ2+ or to 34.7 in cΠ4; the last value corresponds to the diabatic dissociation energy of the cΠ4 state. The bond distances range from 2.513 Å (bΠ4) to 3.354 Å (aΣ8+). Finally, the dipole moment values range from 1.19 D (BΠ6) to 3.72 D (Π2).

The present work highlights the exceptional ability of lithium atoms to participate in a variety of bonding schemes. In the MoLi molecule, the Li atom can participate actively in the formation of bonds by contributing its single valence electron of its ground or first excited atomic state to Mo and/or passively become the recipient of electron density in its vacant orbitals, despite their high energy. The arising bonds vary from van der Waals interactions (aΣ8+) or to as high as two and a half bonds (cΠ4 and Π2). In most cases states, 2s2p_z_ hybridization on Li and 5s4d_0_5p_z_ or 5s5p_z_ hybridization on Mo is found, while electron charge is transferred from Li to Mo up to 0.4 e. Moreover, it is observed that the excited Li(^2^P) atom forms shorted bonds because its empty 2s^0^ orbital can easily accept an electron charge from the 5s4d_0_5p_z_ orbitals of Mo, and thus, a strong σ dative bond can be formed.

As far as we know, the electronic spectra of MoLi have never been studied before, neither experimentally nor theoretically. The binding schemes of all calculated states are presented for the first time, filling the gap in the literature regarding diatomic molecules. Moreover, regarding material and complexes, it was found that, on average, a single covalent Mo-Li bond is about 21 kcal/mol, while if the Mo is excited, on average, a single Mo-Li bond is about 13 kcal/mol. These values can be used to evaluate the interaction energy of the chemically formed Mo-Li bonds in materials and complexes. Finally, the van der Waals interaction is calculated at 2.3 kcal/mol, and this value can be used to evaluate the interaction energy of the physical adsorbed formed Mo…Li bonds in materials with no defect. Even though this energy is low, it shows that Li has the potential to be initially attached to the material. Therefore, this study is useful for both experimentalists and theoreticians, as it could provide the opening gate for further investigation of this species or associated material and complexes.

## Figures and Tables

**Table 1:** Equilibrium bond distances ($R_{e}/Å$) and relative energy differences (T_e_/eV and kcal/mol) for the calculated states of MoLi at the SA-CASSCF/aug-cc-pV5Z(-PP) level of theory.

STATE	*R*_e_	*T*_e_ (eV)	*T*_e_ (kcal/mol)
X ^6^Σ^+^(1)	2.802	0.000	00.00
^8^Σ^+^(1)		(repulsive)	
^8^Π(1)	2.674	1.395	32.2
^6^Σ^+^(2)	2.882	1.577	36.4
^6^Σ^+^(3)	3.004	1.806	41.6
^4^Π(1)	2.620	1.812	41.8
^6^Π(1)	2.982	1.887	43.5
^4^Σ^+^	2.731	1.918	44.2
^4^Δ(1)	2.711	1.977	45.6
^4^Φ	2.656	1.988	45.9
^8^Σ^+^(2)	2.869	2.019	46.6
^6^Δ(1)	3.044	2.036	46.9
^4^Δ(2)	2.756	2.115	48.8
^4^Π(2)	2.726	2.142	49.4
^6^Π(2)	2.873	2.149	49.5
^4^Γ	2.762	2.207	50.9
^4^Σ^−^	2.702	2.434	56.1
^4^Π(3)	2.801	2.455	56.6
^6^Φ	2.712	2.674	61.7
^6^Π(3)	2.892	2.756	63.5
^6^Δ(2)	2.816	2.800	64.6
^6^Σ^−^	2.809	3.003	69.6
^6^Γ		(repulsive)	
^2^Σ^+^(1)	2.663	3.080	71.0
^2^Π(1)	2.634	3.094	71.4
^2^Δ(1)	2.627	3.100	71.5
^2^Σ^+^(2)	2.654	3.119	71.9
^2^Φ	2.657	3.148	72.6
^2^Γ	2.683	3.170	73.1
^2^Π(2)	2.668	3.177	73.3
^8^Φ	3.098	3.183	73.4
^8^Π(2)	3.106	3.220	74.2
^2^Ι	2.705	3.220	74.2
^2^H	2.693	3.220	74.3
^8^Δ(1)	3.225	3.243	74.8
^2^Δ(2)	2.614	3.247	74.9
^8^Π(3)	3.100	3.328	76.7
^8^Σ^−^	3.181	3.412	78.7
^8^Σ^+^(3)		(repulsive)	
^8^Δ(2)	2.650	3.981	91.8
^8^Δ(3)	2.660	4.028	92.9

**Table 2:** Bond distances ($R_{e}$/Å), adiabatic dissociation energies and diabatic in parenthesis ($D_{e}$/kcal/mol), harmonic frequencies ($\omega_{e}$/${\mathrm{cm}}^{-1}$), anharmonic corrections ($\omega_{e}x_{e}$/${\mathrm{cm}}^{-1}$), dipole moments (*μ*/D), and relative energy differences ($T_{e}$/kcal/mol) of the MoLi molecule at the CASSCF, MRCISD, and MRCISD+Q/aug-cc-pV5Z(-PP) levels of theory.

States	Methodology	$R_{e}$	$D_{e}$	$\omega_{e}$	$\omega_{e}x_{e}$	${\mu_{FF}\;}^{a}$	${\langle \mu \rangle \;}^{a}$	$T_{e}$
XΣ+6	CASSCF	2.817	09.66	273.3	2.29	1.87	1.87	00.00
	MRCISD	2.717	22.67	312.1	2.12	3.46	2.69	00.00
	MRCISD+Q	2.708	24.06	316.8	2.11	3.63		00.00
aΣ+8	CASSCF	(repulsive)						
	MRCISD	3.436	01.60	086.7	4.44	1.30	0.64	21.08
	MRCISD+Q	3.354	02.26	101.1	4.06	1.54		21.80
AΣ+6	CASSCF	3.134	14.84	181.2	−1.42	0.50	0.50	40.22
	MRCISD	3.046	17.86	184.3	−0.04	1.24	0.09	36.41
	MRCISD+Q	3.027	19.04	198.8	−2.79	1.42		34.88
bΠ8	CASSCF	2.688	25.80	309.6	1.61	2.30	2.30	32.23
	MRCISD	2.653	29.43	321.9	1.68	2.97	2.80	36.20
	MRCISD+Q	2.654	30.22	321.1	1.72	3.03	--	36.48
cΠ4	CASSCF	2.628	1.74(15.8) ^b^	338.1	1.72	2.18	2.18	49.82
	MRCISD	2.575	18.28(32.3) ^b^	352.7	1.85	3.14	2.96	40.90
	MRCISD+Q	2.570	20.68(34.7) ^b^	354.6	1.85s	3.32		39.35
dΣ+4	CASSCF	2.729	00.42	325.3	2.13	2.30		53.06
	MRCISD	2.678	10.10	338.1	0.70		3.27	43.45
	MRCISD+Q	2.674	11.89	339.5	0.43			41.72
BΠ6	CASSCF	2.766	3.44(9.90) ^c^	248.3	1.94	1.01	1.01	48.12
	MRCISD	2.528	15.38(21.83) ^c^	301.5	1.46	0.89	0.42	43.80
	MRCISD+Q	2.513	17.98(24.66) ^c^	311.7	1.67	1.19		42.05
CΣ6+	CASSCF	2.949	09.38	319.3	11.74	1.04	1.04	50.64
	MRCISD	2.997	13.01	253.2	04.96		0.42	49.21
	MRCISD+Q	3.029	13.24	237.2	02.27			47.71
Π6 (2)	CASSCF	3.027	8.11(1.65) ^d^	267.0	2.83	2.12	2.12	46.43
	MRCISD	2.988	14.11(7.66) ^d^	237.8	4.51		2.86	51.52
	MRCISD+Q	2.981	15.64(8.96) ^d^	230.4	5.11			51.06
Π4 (2)	MRCISD	3.064	19.5(5.50) ^e^	193	8.0		1.70	53.68
	MRCISD+Q	2.954	21.9(7.86) ^e^	217	15.1			52.17
Σ2+	CASSCF	2.682	14.46	340.2	2.05	2.29	2.29	78.63
	MRCISD	2.639	29.42	353.8	1.87	3.56	3.22	62.59
	MRCISD+Q	2.635	31.45	355.7	1.90	3.72		60.31
Π2	CASSCF	2.636	10.09	339.2	2.22	2.64	2.64	80.46
	MRCISD	2.594	25.02	365.9	1.35	3.53	3.35	66.73
	MRCISD+Q	2.589	27.03	370.1	1.23	3.70		64.63

^a^ *μ*_FF_: Dipole moment via finite field; 〈μ〉: dipole moment calculated as an expectation value. ^b^ Adiabatic *D*_e_ with respect to Mo(aD5;5s24d4) + Li(S2); diabatic *D*_e_ with respect to Mo(aG5;5s14d5) + Li(S2) in parenthesis. ^c^ Adiabatic *D*_e_ with respect to Mo(aD5;5s24d4) + Li(S2); diabatic *D*_e_ with respect to Mo(aS7) + Li(P2) in parenthesis. ^d^ Adiabatic *D*_e_ with respect to Mo(aS7) + Li(P2); diabatic *D*_e_ with respect to Mo(aD5;5s24d4) + Li(S2) in parenthesis. ^e^ Adiabatic *D*_e_ with respect to Mo(aG5;5s14d5) + Li(S2); diabatic *D*_e_ with respect to Mo(aD5;5s24d4) + Li(S2) in parenthesis.

**Table 3:** Composition of the molecular orbitals of selected states of MoLi.

State	MOs	LCAOs
XΣ+6	1σ	0.79 $\varphi_{5s}\left(\mathrm{Mo}\right)$ + 0.34 $\varphi_{5p_{z}}\left(\mathrm{Mo}\right)$ + 0.69 $\varphi_{2s}\left(\mathrm{Li}\right)$ − 0.33 $\varphi_{2p_{z}}\left(\mathrm{Li}\right)$
	2σ	0.97 $\varphi_{4d_{z^{2}}}\left(\mathrm{Mo}\right)$
	1$\delta_{+}$	1.00 $\varphi_{4d_{x^{2}-y^{2}}}\left(\mathrm{Mo}\right)$
	1$\pi_{x}$	0.99 $\varphi_{4d_{\mathrm{xz}}}\left(\mathrm{Mo}\right)$
	1$\pi_{y}$	0.99 $\varphi_{4d_{\mathrm{yz}}}\left(\mathrm{Mo}\right)$
	1$\delta_{-}$	1.00 $\varphi_{4d_{\mathrm{xy}}}\left(\mathrm{Mo}\right)$
aΣ+8	1σ	0.12 $\varphi_{5s}\left(\mathrm{Mo}\right)$ + 0.47 $\varphi_{5p_{z}}\left(\mathrm{Mo}\right)$ + 0.98 $\varphi_{2s}\left(\mathrm{Li}\right)$
	2σ	0.98 $\varphi_{5s}\left(\mathrm{Mo}\right)$ − 0.11 $\varphi_{5p_{z}}\left(\mathrm{Mo}\right)$ − 0.11 $\varphi_{2s}\left(\mathrm{Li}\right)$ − 0.18 $\varphi_{2p_{z}}\left(\mathrm{Li}\right)$
	3σ	0.98 $\varphi_{4d_{z^{2}}}\left(\mathrm{Mo}\right)$
	1$\delta_{+}$	1.00 $\varphi_{4d_{x^{2}-y^{2}}}\left(\mathrm{Mo}\right)$
	1$\pi_{x}$	0.99 $\varphi_{4d_{\mathrm{xz}}}\left(\mathrm{Mo}\right)$
	1$\pi_{y}$	0.99 $\varphi_{4d_{\mathrm{yz}}}\left(\mathrm{Mo}\right)$
	1$\delta_{-}$	1.00 $\varphi_{4d_{\mathrm{xy}}}\left(\mathrm{Mo}\right)$
AΣ+6	1σ	0.84 $\varphi_{5s}\left(\mathrm{Mo}\right)$ + 0.24 $\varphi_{5p_{z}}\left(\mathrm{Mo}\right)$ + 0.55 $\varphi_{2s}\left(\mathrm{Li}\right)$ − 0.30 $\varphi_{2p_{z}}\left(\mathrm{Li}\right)$
	2σ	0.95 $\varphi_{4d_{z^{2}}}\left(\mathrm{Mo}\right)$ − 0.19 $\varphi_{5s}\left(\mathrm{Mo}\right)$ + 0.14 $\varphi_{5p_{z}}\left(\mathrm{Mo}\right)$ + 0.23 $\varphi_{2s}\left(\mathrm{Li}\right)$ − 0.11 $\varphi_{2p_{z}}\left(\mathrm{Li}\right)$
	3σ	−0.24 $\varphi_{4d_{z^{2}}}\left(\mathrm{Mo}\right)$ − 0.41 $\varphi_{5s}\left(\mathrm{Mo}\right)$ + 0.40 $\varphi_{5p_{z}}\left(\mathrm{Mo}\right)$ + 0.73 $\varphi_{2s}\left(\mathrm{Li}\right)$ + 0.31 $\varphi_{2p_{z}}\left(\mathrm{Li}\right)$
	1$\delta_{+}$	1.00 $\varphi_{4d_{x^{2}-y^{2}}}\left(\mathrm{Mo}\right)$
	1$\pi_{x}$	0.99 $\varphi_{4d_{\mathrm{xz}}}\left(\mathrm{Mo}\right)$
	1$\pi_{y}$	0.99 $\varphi_{4d_{\mathrm{yz}}}\left(\mathrm{Mo}\right)$
	1$\delta_{-}$	1.00 $\varphi_{4d_{\mathrm{xy}}}\left(\mathrm{Mo}\right)$
BΠ8	1σ	−0.18 $\varphi_{4d_{z^{2}}}\left(\mathrm{Mo}\right)$ + 0.89 $\varphi_{5s}\left(\mathrm{Mo}\right)$ + 0.40 $\varphi_{5p_{z}}\left(\mathrm{Mo}\right)$ + 0.55 $\varphi_{2s}\left(\mathrm{Li}\right)$ − 0.36 $\varphi_{2p_{z}}\left(\mathrm{Li}\right)$
	2σ	0.96 $\varphi_{4d_{z^{2}}}\left(\mathrm{Mo}\right)$ + 0.12 $\varphi_{5s}\left(\mathrm{Mo}\right)$ + 0.27 $\varphi_{5p_{z}}\left(\mathrm{Mo}\right)$ + 0.40 $\varphi_{2s}\left(\mathrm{Li}\right)$ − 0.40 $\varphi_{2p_{z}}\left(\mathrm{Li}\right)$
	1$\delta_{+}$	1.00 $\varphi_{4d_{x^{2}-y^{2}}}\left(\mathrm{Mo}\right)$
	1$\pi_{x}$	0.98 $\varphi_{4d_{\mathrm{xz}}}\left(\mathrm{Mo}\right)$ + 0.17 $\varphi_{2p_{x}}\left(\mathrm{Li}\right)$
	2$\pi_{x}$	−0.14 $\varphi_{4d_{\mathrm{xz}}}\left(\mathrm{Mo}\right)$ + 0.40 $\varphi_{5p_{x}}\left(\mathrm{Mo}\right)$ + 0.59 $\varphi_{2p_{x}}\left(\mathrm{Li}\right)$
	1$\pi_{y}$	0.98 $\varphi_{4d_{\mathrm{yz}}}\left(\mathrm{Mo}\right)$ + 0.17 $\varphi_{2p_{y}}\left(\mathrm{Li}\right)$
	2$\pi_{y}$	−0.14 $\varphi_{4d_{\mathrm{yz}}}\left(\mathrm{Mo}\right)$ + 0.40 $\varphi_{5p_{y}}\left(\mathrm{Mo}\right)$ + 0.59 $\varphi_{2p_{y}}\left(\mathrm{Li}\right)$
	1$\delta_{-}$	1.00 $\varphi_{4d_{\mathrm{xy}}}\left(\mathrm{Mo}\right)$
CΠ4	1σ	0.26 $\varphi_{4d_{z^{2}}}\left(\mathrm{Mo}\right)$ + 0.85 $\varphi_{5s}\left(\mathrm{Mo}\right)$ + 0.30 $\varphi_{5p_{z}}\left(\mathrm{Mo}\right)$ + 0.66 $\varphi_{2s}\left(\mathrm{Li}\right)$ − 0.40 $\varphi_{2p_{z}}\left(\mathrm{Li}\right)$
	1$\delta_{+}$	1.00 $\varphi_{4d_{x^{2}-y^{2}}}\left(\mathrm{Mo}\right)$
	1$\pi_{x}$	0.96 $\varphi_{4d_{\mathrm{xz}}}\left(\mathrm{Mo}\right)$ + 0.27 $\varphi_{2p_{x}}\left(\mathrm{Li}\right)$
	1$\pi_{y}$	0.96 $\varphi_{4d_{\mathrm{yz}}}\left(\mathrm{Mo}\right)$ + 0.27 $\varphi_{2p_{y}}\left(\mathrm{Li}\right)$
	1$\delta_{-}$	1.00 $\varphi_{4d_{\mathrm{xy}}}\left(\mathrm{Mo}\right)$
dΣ+4	1σ	0.26 $\varphi_{4d_{z^{2}}}\left(\mathrm{Mo}\right)$ + 0.80 $\varphi_{5s}\left(\mathrm{Mo}\right)$ + 0.34 $\varphi_{5p_{z}}\left(\mathrm{Mo}\right)$ + 0.69 $\varphi_{2s}\left(\mathrm{Li}\right)$ − 0.40 $\varphi_{2p_{z}}\left(\mathrm{Li}\right)$
	2σ	0.87 $\varphi_{4d_{z^{2}}}\left(\mathrm{Mo}\right)$ − 0.30 $\varphi_{5s}\left(\mathrm{Mo}\right)$ + 0.18 $\varphi_{5p_{z}}\left(\mathrm{Mo}\right)$ + 0.14 $\varphi_{2s}\left(\mathrm{Li}\right)$ − 0.21 $\varphi_{2p_{z}}\left(\mathrm{Li}\right)$
	1$\delta_{+}$	1.00 $\varphi_{4d_{x^{2}-y^{2}}}\left(\mathrm{Mo}\right)$
	1$\pi_{x}$	0.98 $\varphi_{4d_{\mathrm{xz}}}\left(\mathrm{Mo}\right)$ + 0.23 $\varphi_{2p_{x}}\left(\mathrm{Li}\right)$
	1$\pi_{y}$	0.98 $\varphi_{4d_{\mathrm{yz}}}\left(\mathrm{Mo}\right)$ + 0.23 $\varphi_{2p_{y}}\left(\mathrm{Li}\right)$
	1$\delta_{-}$	1.00 $\varphi_{4d_{\mathrm{xy}}}\left(\mathrm{Mo}\right)$
BΠ6	1σ	0.72 $\varphi_{4d_{z^{2}}}\left(\mathrm{Mo}\right)$ + 0.64 $\varphi_{5s}\left(\mathrm{Mo}\right)$ + 0.41 $\varphi_{5p_{z}}\left(\mathrm{Mo}\right)$ + 0.65 $\varphi_{2s}\left(\mathrm{Li}\right)$ − 0.53 $\varphi_{2p_{z}}\left(\mathrm{Li}\right)$
	2σ	0.67 $\varphi_{4d_{z^{2}}}\left(\mathrm{Mo}\right)$ − 0.66 $\varphi_{5s}\left(\mathrm{Mo}\right)$ − 0.12 $\varphi_{5p_{z}}\left(\mathrm{Mo}\right)$ − 0.24 $\varphi_{2s}\left(\mathrm{Li}\right)$
	1$\delta_{+}$	1.00 $\varphi_{4d_{x^{2}-y^{2}}}\left(\mathrm{Mo}\right)$
	1$\pi_{x}$	0.91 $\varphi_{4d_{\mathrm{xz}}}\left(\mathrm{Mo}\right)$ + 0.40 $\varphi_{2p_{x}}\left(\mathrm{Li}\right)$
	1$\pi_{y}$	0.91 $\varphi_{4d_{\mathrm{yz}}}\left(\mathrm{Mo}\right)$ + 0.40 $\varphi_{2p_{y}}\left(\mathrm{Li}\right)$
	1$\delta_{-}$	1.00 $\varphi_{4d_{\mathrm{xy}}}\left(\mathrm{Mo}\right)$
CΣ+6	1σ	0.80 $\varphi_{5s}\left(\mathrm{Mo}\right)$ + 0.30 $\varphi_{5p_{z}}\left(\mathrm{Mo}\right)$ + 0.29 $\varphi_{4d_{z^{2}}}\left(\mathrm{Mo}\right)$ + 0.57 $\varphi_{2s}\left(\mathrm{Li}\right)$ − 0.30 $\varphi_{2p_{z}}\left(\mathrm{Li}\right)$
	2σ	−0.34 $\varphi_{5s}\left(\mathrm{Mo}\right)$ + 0.48 $\varphi_{5p_{z}}\left(\mathrm{Mo}\right)$ − 0.44 $\varphi_{4d_{z^{2}}}\left(\mathrm{Mo}\right)$ + 0.66 $\varphi_{2s}\left(\mathrm{Li}\right)$
	1$\delta_{+}$	1.00 $\varphi_{4d_{x^{2}-y^{2}}}\left(\mathrm{Mo}\right)$
	1$\pi_{x}$	0.99 $\varphi_{4d_{\mathrm{xz}}}\left(\mathrm{Mo}\right)$
	1$\pi_{y}$	0.99 $\varphi_{4d_{\mathrm{yz}}}\left(\mathrm{Mo}\right)$
	1$\delta_{-}$	1.00 $\varphi_{4d_{\mathrm{xy}}}\left(\mathrm{Mo}\right)$
Σ2+	1σ	0.38 $\varphi_{4d_{z^{2}}}\left(\mathrm{Mo}\right)$ + 0.78 $\varphi_{5s}\left(\mathrm{Mo}\right)$ + 0.36 $\varphi_{5p_{z}}\left(\mathrm{Mo}\right)$ + 0.70 $\varphi_{2s}\left(\mathrm{Li}\right)$ − 0.44 $\varphi_{2p_{z}}\left(\mathrm{Li}\right)$
	2σ	0.89 $\varphi_{4d_{z^{2}}}\left(\mathrm{Mo}\right)$ − 0.44 $\varphi_{5s}\left(\mathrm{Mo}\right)$ + 0.13 $\varphi_{5p_{z}}\left(\mathrm{Mo}\right)$
	1$\delta_{+}$	1.00 $\varphi_{4d_{x^{2}-y^{2}}}\left(\mathrm{Mo}\right)$
	1$\pi_{x}$	0.95 $\varphi_{4d_{\mathrm{xz}}}\left(\mathrm{Mo}\right)$ + 0.17 $\varphi_{5p_{x}}\left(\mathrm{Mo}\right)$ + 0.28 $\varphi_{2p_{x}}\left(\mathrm{Li}\right)$
	1$\pi_{y}$	0.95 $\varphi_{4d_{\mathrm{yz}}}\left(\mathrm{Mo}\right)$ + 0.17 $\varphi_{5p_{y}}\left(\mathrm{Mo}\right)$ + 0.28 $\varphi_{2p_{y}}\left(\mathrm{Li}\right)$
	1$\delta_{-}$	1.00 $\varphi_{4d_{\mathrm{xy}}}\left(\mathrm{Mo}\right)$
Π2	1σ	0.24 $\varphi_{4d_{z^{2}}}\left(\mathrm{Mo}\right)$ + 0.85 $\varphi_{5s}\left(\mathrm{Mo}\right)$ + 0.30 $\varphi_{5p_{z}}\left(\mathrm{Mo}\right)$ + 0.66 $\varphi_{2s}\left(\mathrm{Li}\right)$ − 0.41$\varphi_{2p_{z}}\left(\mathrm{Li}\right)$
	1$\delta_{+}$	1.00 $\varphi_{4d_{x^{2}-y^{2}}}\left(\mathrm{Mo}\right)$
	1$\pi_{x}$	0.96 $\varphi_{4d_{\mathrm{xz}}}\left(\mathrm{Mo}\right)$ + 0.27 $\varphi_{2p_{x}}\left(\mathrm{Li}\right)$
	1$\pi_{y}$	0.96 $\varphi_{4d_{\mathrm{yz}}}\left(\mathrm{Mo}\right)$ + 0.27 $\varphi_{2p_{y}}\left(\mathrm{Li}\right)$
	1$\delta_{-}$	1.00 $\varphi_{4d_{\mathrm{xy}}}\left(\mathrm{Mo}\right)$

**Table 4:** Equilibrium products and asymptotic products of selected states of MoLi.

State	Equilibrium Products	Asymptotic Products
XΣ+6 , aΣ+8	Mo (aS7 ) + Li (S2 )	Mo (aS7 ) + Li (S2 )
AΣ+6	Mo (aS5 ) + Li (S2 )	Mo (aS5 ) + Li (S2 )
BΠ6	Mo (aS7 ) + Li (P2 )	Mo (aD5 ) + Li (P2 )
CΣ+6	Mo (aD5 ) + Li (S2 )	Mo (aD5 ) + Li (S2 )
bΠ8	Mo (aS7 ) + Li (P2 )	Mo (aS7 ) + Li (P2 )
cΠ4	Mo (aG5 ) + Li (S2 )	Mo (aD5 ) + Li (S2 )
Π6 (2)	Mo (aD5 ) + Li (P2 )	Mo (aS7 ) + Li (P2 )
Π4 (2)	Mo (aD5 ) + Li (S2 )	Mo (aG5 ) + Li (S2 )
DΣ+4	Mo (aS5 ) + Li (S2 )	Mo (aS5 ) + Li (S2 )
Σ2+ , Π2	Mo (aD3oraG3 ) + Li (S2 )	Mo (aD3oraG3 ) + Li (S2 )

## Data Availability

Data are contained within the article and Supplementary Materials.

## Supplementary Materials

The following supporting information can be downloaded at: https://www.mdpi.com/article/10.3390/molecules30132874/s1. Energetics and potential energy curves of the ground and excited states of MoLi at CASSCF, MRCISD, and MRCISD+Q/aug-cc-pV5Z(-PP) levels of theory.

## References

[1] Stiefel E.I. (2001). Kirk-Othmer Encyclopedia of Chemical Technology.

[2] Pan F., Huang D., Li Y., Yuan X., Cao Y. (2009). Crystal structure and magnetic properties of Li,Cr-containing molybdates Li_3_Cr(MoO_4_)_3_, LiCr(MoO_4_)_2_ and Li_1.8_Cr_1.2_(MoO_4_)_3_. J. Solid State Chem..

[3] Liu Y., Lin Z., Bettels F., Li Z., Xu J., Zhang Y., Li X., Ding F., Liu S., Zhang L. (2023). Molybdenum-Based Catalytic Materials for Li–S Batteries: Strategies, Mechanisms, and Prospects. Adv. Energy Sustain. Res..

[4] Shon J.K., Lee H.S., Park G.O., Yoon J., Park E., Park G.S., Kong S.S., Jin M., Choi J.-M., Chang H. (2016). Discovery of abnormal lithium-storage sites in molybdenum dioxide electrodes. Nat. Commun..

[5] Su Q., Wang S., Feng M., Du G., Xu B. (2017). Direct Studies on the Lithium-Storage Mechanism of Molybdenum Disulfide. Sci. Rep..

[6] Huang Y., Field R., Chen Q., Peng Y., Walczak M.S., Zhao H., Zhu G., Liu Z., Li L. (2019). Laser induced molybdenum sulphide loading on doped graphene cathode for highly stable lithium sulphur battery. Commun. Chem..

[7] Wan J., Hao Y., Shi Y., Song Y.-X., Yan H.-J., Zheng J., Wen R., Wan L.-J. (2019). Ultra-thin solid electrolyte interphase evolution and wrinkling processes in molybdenum disulfide-based lithium-ion batteries. Nat. Commun..

[8] Gao D., Deng S., Chen X., Zhang Y., Lv T., He Y., Zhou F., Zhang W., Chu P.K., Huo K. (2023). Mixed Ion/Electron Conductive Li_3_N–Mo Interphase Enabling Stable and Ultrahigh-Rate Lithium Metal Anodes. ACS Appl. Mater. Interfaces.

[9] Chen M., Roszell J., Scoullos E.V., Riplinger C., Koel B.E., Carter E.A. (2016). Effect of Temperature on the Desorption of Lithium from Molybdenum(110) Surfaces: Implications for Fusion Reactor First Wall Materials. J. Phys. Chem. B.

[10] Zhang Y., Liu H., Zhang L., Wu L., Liu Y., Wang Z., Zhang Y., Zhao Y., Ren Y., Chen Y. (2021). Molybdenum Host and Interphase Induced Decentralized Lithium Deposition for Dendrite-Free Lithium Metal Anodes. Energy Storage Mater..

[11] Ma C., Wang Z., Wang H., Chen J., Zhang Q., Shen J., Han W., Yang W. (2021). Strong and Stable Metal–Organic Framework Membrane with Engineered Macro–Micro Hierarchically Structured Channels for Hydrogen Purification. ACS Nano.

[12] Jiang H., Gu S., Guo J., Dai Y., Zheng W., Jiang X., Wu X., Xiao W., He G., Li X. (2022). Position Difference between Mo Clusters and N Sites Induced Highly Synergistic Electrocatalysis in Integrated Electrode-Separator Membranes with Crosslinked Hierarchically Porous Interface. Energy Storage Mater..

[13] Li Z., Sami I., Yang J., Li J., Kumar R.V., Chhowalla M. (2023). Lithiated metallic molybdenum disulfide nanosheets for high-performance lithium–sulfur batteries. Nat. Energy.

[14] Harrison J.F. (2000). Electronic structure of diatomic molecules composed of a first-row transition metal and main-group element (h-f). Chem. Rev..

[15] Mermigki M.A., Karapetsas I., Tzeli D. (2023). Electronic structure of low-lying states of triatomic MoS_2_ molecule. The building block of 2D MoS2. Chem. Phys. Chem..

[16] Tzeli D., Karapetsas I., Merriles D.M., Ewigleben J.C., Morse M.D. (2022). The molybdenum-sulfur bond: Electronic structure of low-lying states of MoS. J. Phys. Chem. A.

[17] Hoffman B.M., Lukoyanov D., Yang Z.-Y., Dean D.R., Seefeldt L.C. (2014). Mechanism of nitrogen fixation by nitrogenase: The next stage. Chem. Rev..

[18] White M.V., Claveau E.E., Miliordos E., Vogiatzis K.D. (2024). Electronic Structure and Ligand Effects on the Activation and Cleavage of N_2_ on a Molybdenum Center. J. Phys. Chem. A.

[19] Sorensen J.J., Tieu E., Sevy A., Merriles D.M., Nielson C., Ewigleben J.C., Morse M.D. (2020). Bond dissociation energies of transition metal oxides: CrO, MoO, RuO, and RhO. J. Chem. Phys..

[20] Zhang L., Zou W., Yu Y., Zhao D., Maa X., Yang J. (2021). Spin-orbit splittings in the low-lying states of MoO molecule. J. Quant. Spectrosc. Radiat. Transf..

[21] Depastas T., Androutsopoulos A., Tzeli D. (2022). Analysis of chemical bonding of the ground and low-lying states of Mo_2_ and of Mo_2_Cl_x_ complexes, x = 2-10. J. Chem. Phys..

[22] Demetriou C., Tzeliou C.E., Androutsopoulos A., Tzeli D. (2023). Electronic Structure and Chemical Bonding of the First-, Second-, and Third-Row-Transition-Metal Monoborides: The Formation of Quadruple Bonds in RhB, RuB, and TcB. Molecules.

[23] Androutsopoulos A., Tzeli D., Tomchak K.H., Morse M.D. (2024). Quadruple bonds in MoC: Accurate calculations and precise measurement of the dissociation energy of low-lying states of MoC. J. Chem. Phys..

[24] Tzeli D., Papakondylis A., Mavridis A. (1997). On the Electronic Structure of the Ground (X^3^Σ^-^) and Some Low-Lying States (A^3^Π, a^1^Δ, b^1^Σ^+^, B^3^Σ^-^) of the Isovalent Species P-Li and P-Na. Mol. Struct. (THEOCHEM).

[25] Tzeli D., Papakondylis A., Mavridis A. (1998). On the Electronic Structure of NLi_2_ and PLi_2_. Ground and Low-Lying Excited States. J. Phys. Chem. A.

[26] Sanli A., Pan X., Beecher D.S., Magnier S., Lyyra A.M., Ahmed E.H. (2019). Electronic transition dipole moment and radiative lifetime calculations of lithium dimer ion-pair states. J. Mol. Spectr..

[27] Androutsopoulos A., Tzeli D. (2025). Electronic structure and chemical bonding of MoX molecules, where X = Li, Be, B, C, N, O, and F. ACS Omega.

[28] Sugar J., Musgrove A. (1988). Energy Levels of Molybdenum, Mo I through Mo XLII. J. Phys. Chem. Ref. Data.

[29] Kramida A., Ralchenko Y., Reader J., Olsen K., Ibacache R. NIST Standard Reference Database 78, Version 5.12. https://www.nist.gov/pml/atomic-spectra-database.

[30] Finelli S., Ciamei A., Restivo B., Schemmer M., Cosco A., Inguscio M., Trenkwalder A., Zaremba-Kopczyk K., Gronowski M., Tomza M. (2024). Ultracold LiCr: A New Pathway to Quantum Gases of Paramagnetic Polar Molecules. PRX Quantum.

[31] Lawson D.B., Harrison J.F. (1996). Electronic Structures of ScLi, TiLi, VLi, CrLi, and CuLi and Their Positive Ions. J. Phys. Chem. A.

[32] Borin A.C., Gobbo J.P. (2011). Electronic Structure of the Ground and Low-Lying Electronic States of MoB and MoB⁺. Int. J. Quantum Chem..

[33] Pérez-Jiménez M., Campos J., Jover J., Álvarez S., Carmona E. (2015). Supported σ-Complexes of Li−C Bonds from Coordination of Monomeric Molecules of LiCH_3_, LiCH_2_CH_3_ and LiC_6_H_5_ to Mo≣Mo Bonds. Angew. Chem. Int. Ed..

[34] Perez-Jimenez M., Curado N., Maya C., Campos J., Jover J., Alvarez S., Carmona E. (2021). Coordination of LiH Molecules to Mo≣Mo Bonds: Experimental and Computational Studies on Mo2LiH2, Mo2Li2H4, and Mo6Li9H18 Clusters. J. Am. Chem. Soc..

[35] Liu S.-C., Ke W.-L., Yu J.-S.K., Kuo T.-S., Tsa Y.-C. (2012). An Electron-Rich Molybdenum–Molybdenum Quintuple Bond Spanned by One Lithium Atom. Angew. Chem. Int. Ed..

[36] Sun X., Wang Z., Fu Y.Q. (2015). Defect-Mediated Lithium Adsorption and Diffusion on Monolayer Molybdenum Disulfide. Sci. Rep..

[37] Ersan F., Gökoğlu G., Aktürk E. (2015). Adsorption and Diffusion of Lithium on Monolayer Transition Metal Dichalcogenides (MoS2(1–x)Se2x) Alloys. J. Phys. Chem. C.

[38] Vincent R.C., Cheetham A.K., Seshadri R. (2023). Structure and lithium insertion in oxides of molybdenum. APL Mater..

[39] Wenhao L., Shaozhen H., Yu Z., Kecheng L., Piao Q., Yaqin W., Shengli A., Zhibin W., Libao C. (2024). Molybdenum dialkyphosphorodithioate-derived artificial solid-electrolyte interface enabling stable lithium metal anodes. Energy Storage Mater..

[40] Zhou Y.G., Zu X.T., Nie J.L., Xiao H.Y. (2009). Adsorption of Li on Mo(110) Surface: A First-Principles Study. Surf. Rev. Lett..

[41] Tsendin D.V., Dufek T., Yu J.H., Maroudas D. (2020). Characterization of the Liquid Li–Solid Mo (110) Interface from Classical Molecular Dynamics for Plasma-Facing Applications. J. Nucl. Mater..

[42] Peterson K.A., Figgen D., Dolg M., Stoll H. (2007). Energy-consistent relativistic pseudopotentials and correlation consistent basis sets for the elements Y–Pd. J. Chem. Phys..

[43] Prascher B.P., Woon D.E., Peterson K.A., Dunning T.H., Wilson A.K. (2011). Gaussian basis sets for use in correlated molecular calculations. VII. Valence, core-valence, and scalar relativistic basis sets for Li, Be, Na, and Mg. Theor. Chem. Acc..

[44] Shim I., Gingerich K.A. (1997). Electronic states and nature of bonding in the molecule MoC by all electron ab initio calculations. J. Chem. Phys..

[45] Werner H.-J., Reinsch E.A. (1982). The self-consistent electron pairs method for multiconfiguration reference state functions. J. Chem. Phys..

[46] Werner H.-J. (1987). The Complete Active Space Self-Consistent Field Method and its Applications in Electronic Structure Calculations. Adv. Chem. Phys..

[47] Knowles P.J., Werner H.-J. (1988). An efficient method for the evaluation of coupling coefficients in configuration interaction calculations. Chem. Phys. Lett..

[48] Werner H.-J., Knowles P.J. (1988). An efficient internally contracted multiconfiguration-reference configuration interaction method. J. Chem. Phys..

[49] Langhoff S.R., Davidson E.R. (1974). Configuration interaction calculations on the nitrogen molecule. Int. J. Quantum Chem..

[50] Blomberg M.R.A., Siegbahn P.E.M. (1983). Singlet and triplet energy surfaces of NiH_2_. J. Chem. Phys..

[51] Dunham J.L. (1932). The Wentzel-Brillouin-Kramers Method of Solving the Wave Equation. Phys. Rev..

[52] Dennington R., Keith T.A., Millam J.M. (2016). GaussView, version 6.1.1.

[53] Ishikawa S., Madjarova G., Yamabe T. (2001). First-Principles Study of the Lithium Interaction with Polycyclic Aromatic Hydrocarbons. J. Phys. Chem. B.

[54] Tzeli D., Petsalakis I., Theodorakopoulos G. (2011). Computational Insight into the Electronic Structure and Absorption Spectra of Lithium Complexes of N-confused Tetraphenylporphyrin. J. Phys. Chem. A.

[55] Werner H.-J., Knowles P.J., Manby F.R., Black J.A., Doll K., Heßelmann A., Kats D., Köhn A., Korona T., Kreplin D.A. (2020). MOLPRO version 2022. Chem. Phys..

