# Supplementary material for: Electronic Structure of the Ground and Low-Lying States of MoLi
Source: Molecules. 2025 Jul 6;30(13):2874. doi: 10.3390/molecules30132874 (PMC12250845; doi:10.3390/molecules30132874)
Supplement: Supplementary file 1 [file molecules-30-02874-s001.zip › molecules-3723676-supplementary.pdf]

Supporting Information

Electronic Structure of the ground and low-lying states of MoLi

Constantinos Demetriou[1] and Demeter Tzeli,* [1,2]

[1] *Laboratory of Physical Chemistry, Department of Chemistry, National and Kapodistrian University of Athens, Athens, Greece*

[2] *Theoretical and Physical Chemistry Institute, National Hellenic Research Foundation, Athens, Greece*

Corresponding author: tzeli@chem.uoa.gr

TABLE S1: Equilibrium bond distances (R_e/Å), energy minimums (E/E_h), and relative energy differences (with respect to the lowest-lying electronic state) (T_e/E_h, *e*V, cm^{-1}, and kcal/mol, respectively) for each of all calculated states of MoLi at the CASSCF/AV5Z level of theory.

**STATE**	$\boldsymbol{R_e}$	$\boldsymbol{E}$			$\boldsymbol{T_e}$	
$^2\Sigma^+(1)$	2.663	-74.673612	0.113177	3.080	24839	71.02
$^2\Pi(1)$	2.634	-74.673073	0.113716	3.094	24958	71.36
$^2\Delta(1)$	2.627	-74.672846	0.113943	3.100	25008	71.50
$^2\Sigma^+(2)$	2.654	-74.672158	0.114631	3.119	25158	71.93
$^2\Phi$	2.657	-74.671091	0.115698	3.148	25393	72.60
$^2\Gamma$	2.683	-74.670310	0.116479	3.170	25564	73.09
$^2\Pi(2)$	2.668	-74.670037	0.116752	3.177	25624	73.26
^{2}H	2.693	-74.668436	0.118353	3.220	25975	74.27
^{2}I	2.705	-74.668459	0.118330	3.220	25970	74.25
$^2\Delta(2)$	2.614	-74.667478	0.119311	3.247	26186	74.87
$^4\Pi(1)$	2.620	-74.720208	0.066581	1.812	14613	41.78
$^4\Sigma^+$	2.731	-74.716285	0.070504	1.918	15474	44.24
$^4\Delta(1)$	2.711	-74.714142	0.072647	1.977	15944	45.59
$^4\Phi$	2.656	-74.713712	0.073077	1.988	16039	45.86
$^4\Delta(2)$	2.756	-74.709055	0.077734	2.115	17061	48.78
$^4\Pi(2)$	2.726	-74.708051	0.078738	2.142	17281	49.41
$^4\Gamma$	2.762	-74.705682	0.081107	2.207	17801	50.90
$^4\Sigma^-$	2.702	-74.697353	0.089436	2.434	19629	56.12
$^4\Pi(3)$	2.801	-74.696583	0.090206	2.455	19798	56.60
$\boldsymbol{^6\Sigma^+(1)}$	**2.802**	**-74.786789**	**0.000000**	**0.000**	**0**	**0.00**
$^6\Sigma^+(2)$	2.882	-74.728824	0.057965	1.577	12722	36.37
$^6\Sigma^+(3)$	3.004	-74.720424	0.066365	1.806	14565	41.64
$^6\Pi(1)$	2.982	-74.717432	0.069357	1.887	15222	43.52
$^6\Delta(1)$	3.044	-74.711979	0.074810	2.036	16419	46.94
$^6\Pi(2)$	2.873	-74.707820	0.078970	2.149	17332	49.55
$^6\Phi$	2.712	-74.688535	0.098254	2.674	21564	61.66
$^6\Pi(3)$	2.892	-74.685526	0.101263	2.756	22225	63.54
$^6\Delta(2)$	2.816	-74.683873	0.102916	2.800	22588	64.58
$^6\Sigma^-$	2.809	-74.676423	0.110366	3.003	24223	69.26
$^6\Gamma$[a]	--	Repulsive				
$^8\Sigma^+(1)$[a]	--	Repulsive				
$^8\Pi(1)$	2.674	-74.735513	0.051276	1.395	11254	32.18
$^8\Sigma^+(2)$	2.869	-74.712599	0.074190	2.019	16283	46.56
$^8\Phi$	3.098	-74.669824	0.116964	3.183	25671	73.40
$^8\Pi(2)$	3.106	-74.668464	0.118325	3.220	25969	74.25
$^8\Delta(1)$	3.225	-74.667593	0.119196	3.243	26161	74.80
$^8\Pi(3)$	3.100	-74.664474	0.122315	3.328	26845	76.75
$^8\Sigma^-$	3.181	-74.661414	0.125375	3.412	27517	78.67
$^8\Sigma^+(3)$[a]	--	Repulsive				
$^8\Delta(2)$	2.650	-74.640494	0.146294	3.981	32108	91.80
$^8\Delta(3)$	2.660	-74.638773	0.148016	4.028	32486	92.88

[a] at the CASSCF/AV5Z level these states are repulsive, the reported values correspond, arbitrary,

TABLE S2: Bond distances (R_e/Å), absolute energies (E/E_h), relative energy differences (T_e/kcal/mol and cm^{-1} respectively), adiabatic dissociation energies (D_e/kcal/mol), harmonic frequencies (ω_e/cm^{-1}), anharmonic corrections ($\omega_e x_e$/cm^{-1}), and dipole moments (μ/D) of every state of MoLi at all used levels of theory.

States	Methodology	R_e	E	T_e	T_e	D_e	ω_e	$\omega_e x_e$	μ_{FF} [a]	$\langle\mu\rangle$ [a]
$X\,^6\Sigma^+$	CASSCF	2.817	-74.801770	0.00	0.0	9.66	273.3	2.29	1.87	
	MRCISD	2.717	-74.905182	0.00	0.0	22.67	312.1	2.12	3.46	2.69
	MRCISD+Q	2.708	-74.910722	0.00	0.0	24.06	316.8	2.11	3.63	--
$a\,^8\Sigma^+$	CASSCF	--	--	--	--	--	--	--	--	
	MRCISD	3.436	-74.871597	21.08	7371.1	1.60	86.7	4.44	1.30	0.64
	MRCISD+Q	3.354	-74.875976	21.80	7625.7	2.26	101.1	4.06	1.54	--
$A\,^6\Sigma^+$	CASSCF	3.134	-74.737667	40.22	14069.0	14.84	181.2	1.42	0.50	
	MRCISD	3.046	-74.847153	36.41	12735.8	17.86	184.3	-0.04	1.24	0.09
	MRCISD+Q	3.027	-74.855140	34.88	12198.7	19.04	198.8	2.79	1.42	--
$^8\Pi$	CASSCF	2.688	-74.750412	32.23	11271.8	25.80	309.6	1.61	2.30	
	MRCISD	2.653	-74.847487	36.20	12662.6	29.43	321.9	1.68	2.97	2.80
	MRCISD+Q	2.654	-74.852579	36.48	12761.0	30.22	321.1	1.72	3.03	--
$b\,^4\Pi$	CASSCF	2.628	-74.722383	49.82	17423.5	01.74(15.8)[b]	338.1	1.72	2.18	
	MRCISD	2.575	-74.839997	40.90	14306.5	18.28(32.3)[b]	352.7	1.85	3.14	2.96
	MRCISD+Q	2.570	-74.848012	39.35	13763.3	20.68(34.7)[b]	354.6	1.85s	3.32	--
$c\,^4\Sigma^+$	CASSCF	2.729	-74.717208	53.06	18559.3	0.42	325.3	2.13	2.30	
	MRCISD	2.678	-74.835944	43.45	15196.0	10.10	338.1	0.70	--	3.27
	MRCISD+Q	2.674	-74.844237	41.72	14591.8	11.89	339.5	0.43	--	--
$B\,^6\Pi$	CASSCF	2.766	-74.725080	48.12	16831.6	3.44(9.90)[c]	248.3	1.94	-1.01	
	MRCISD	2.528	-74.835377	43.80	15320.4	15.38(21.83)[c]	301.5	1.46	0.89	0.42
	MRCISD+Q	2.513	-74.843710	42.05	14707.3	17.98(24.66)[c]	311.7	1.67	1.19	--
$C\,^6\Sigma^+$	CASSCF	2.949	-74.721066	50.64	17712.7	9.38	319.3	11.74	1.04	
	MRCISD	2.997	-74.826762	49.21	17211.2	13.01	253.2	4.96	--	0.42
	MRCISD+Q	3.029	-74.834697	47.71	16685.6	13.24	237.2	2.27	--	--
$^6\Pi(2)$	CASSCF	3.027	-74.7277742	46.43	16240	8.11(1.65)	267.0	2.83	2.12	
	MRCISD	2.988	-74.8230790	51.52	18020	14.11(7.66)	237.8	4.51		2.86
	MRCISD+Q	2.981	-74.8293461	51.06	17860	15.64(8.96)	230.4	5.11		
$^4\Pi(2)$	MRCISD	3.064	-74.8196350	53.68	18775	19.5(5.50)	193	8.0		1.70
	MRCISD+Q	2.954	-74.8275820	52.17	18247	21.9(7.86)	217	15.1		

$d\,^2\Sigma^+$	CASSCF	2.682	-74.676468	78.63	27500.8	14.46	340.2	2.05	2.29	
	MRCISD	2.639	-74.805433	62.59	21892.3	29.42	353.8	1.87	3.56	3.22
	MRCISD+Q	2.635	-74.814616	60.31	21092.8	31.45	355.7	1.90	3.72	--
$e\,^2\Pi$	CASSCF	2.636	-74.673556	80.46	28139.7	10.09	339.2	2.22	2.64	
	MRCISD	2.594	-74.798845	66.73	23338.4	25.02	365.9	1.35	3.53	3.35
	MRCISD+Q	2.589	-74.807734	64.63	22603.2	27.03	370.1	1.23	3.70	--

[a] μ_{FF}: Dipole moment via finite field; <μ>: dipole moment calculated as expectation value.

[b] Adiabatic D_e with respect Mo($a\,^5$D; $5s^24d^4$) + Li(^{2}S); diabatic D_e with respect to Mo($a\,^5$G; $5s^14d^5$) + Li(^{2}S) in parenthesis.

[c] Adiabatic D_e with respect Mo($a\,^5$D; $5s^24d^4$) + Li(^{2}S); diabatic D_e with respect to Mo($a\,^7$S) + Li(^{2}P) in parenthesis.

TABLE S3: Configuration State Functions of $^2\Sigma^+(1)$ state.

STATE	COEFFICIENT	CSF OF REFERENCE
$\vert ^2\Sigma^+(1)\rangle$	0.5136993	$\vert 1\sigma^2 2\sigma^1 1\delta_+^1 \overline{1\pi}_x^1 \overline{1\pi}_y^1 1\delta_-^1\rangle$
	-0.3247399	$\vert 1\sigma^2 2\sigma^1 1\delta_+^1 \overline{1\pi}_x^1 1\pi_y^1 \overline{1\delta}_-^1\rangle$
	-0.2296258	$\vert 1\sigma^2 2\sigma^1 1\delta_+^1 1\pi_x^1 \overline{1\pi}_y^1 \overline{1\delta}_-^1\rangle$
	-0.0834114	$\vert 1\sigma^1 2\sigma^2 \overline{1\delta}_+^1 1\pi_x^1 1\pi_y^1 \overline{1\delta}_-^1\rangle$
	0.2874308	$\vert 1\sigma^2 2\sigma^2 1\delta_+^0 1\pi_x^1 \overline{1\pi}_y^1 1\delta_-^1\rangle$
	-0.2175189	$\vert 1\sigma^2 2\sigma^0 1\delta_+^2 1\pi_x^1 \overline{1\pi}_y^1 1\delta_-^1\rangle$
	-0.2032437	$\vert 1\sigma^2 2\sigma^2 1\delta_+^1 1\pi_x^0 1\pi_y^2 1\delta_-^0\rangle$
	0.2032437	$\vert 1\sigma^2 2\sigma^2 1\delta_+^1 1\pi_x^2 1\pi_y^0 1\delta_-^0\rangle$
	-0.1994316	$\vert 1\sigma^2 2\sigma^1 1\delta_+^0 1\pi_x^0 1\pi_y^2 1\delta_-^2\rangle$
	-0.1994316	$\vert 1\sigma^2 2\sigma^1 1\delta_+^0 1\pi_x^2 1\pi_y^0 1\delta_-^2\rangle$
	-0.1994315	$\vert 1\sigma^2 2\sigma^1 1\delta_+^2 1\pi_x^0 1\pi_y^2 1\delta_-^0\rangle$
	-0.1994315	$\vert 1\sigma^2 2\sigma^1 1\delta_+^2 1\pi_x^2 1\pi_y^0 1\delta_-^0\rangle$
	0.1538088	$\vert 1\sigma^2 2\sigma^0 1\delta_+^1 1\pi_x^0 1\pi_y^2 1\delta_-^2\rangle$
	-0.1538088	$\vert 1\sigma^2 2\sigma^0 1\delta_+^1 1\pi_x^2 1\pi_y^0 1\delta_-^2\rangle$
	-0.0584445	$\vert 1\sigma^1 2\sigma^2 1\delta_+^0 1\pi_x^2 1\pi_y^2 1\delta_-^0\rangle$

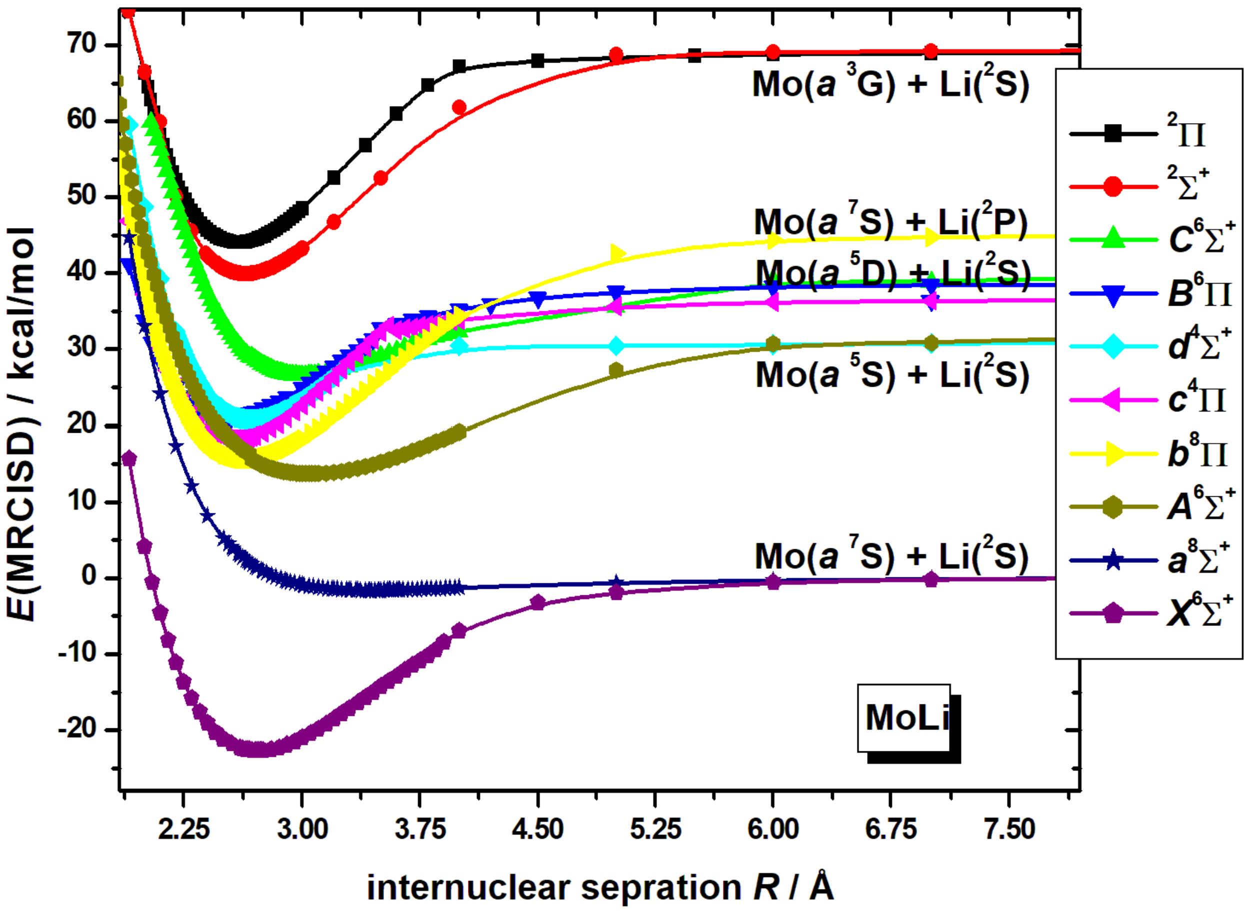

FIGURE S1: Potential Energy Curves of the selected states of MoLi at the MRCISD/aug-cc-pV5Z(-PP) computational level, with respect to the adiabatic asymptotic atomic products of the ground state.

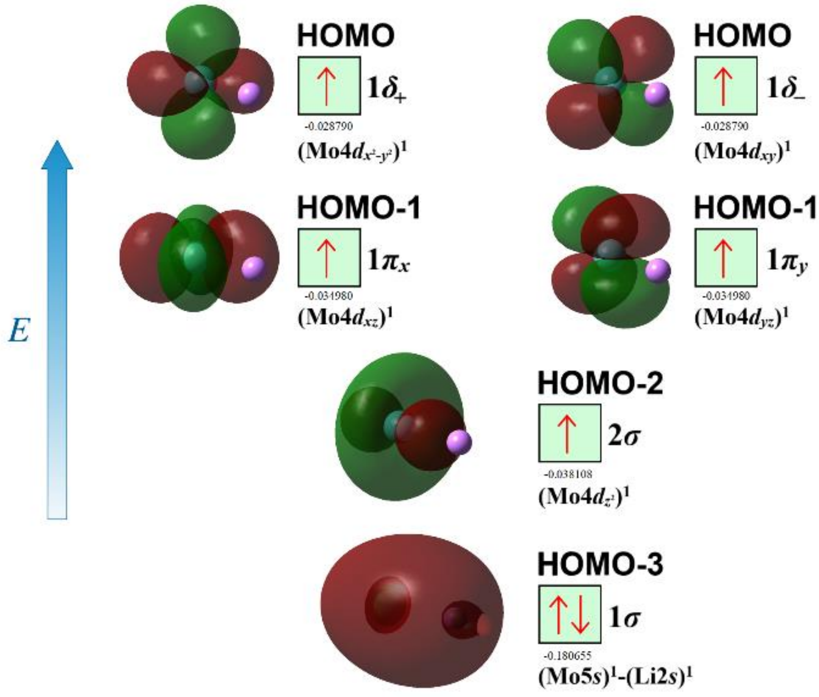

SCHEME S1: MOs energy diagrams of the ground state $X\,^6\Sigma^+(1)$ of MoLi at the CASSCF/AV5Z level.

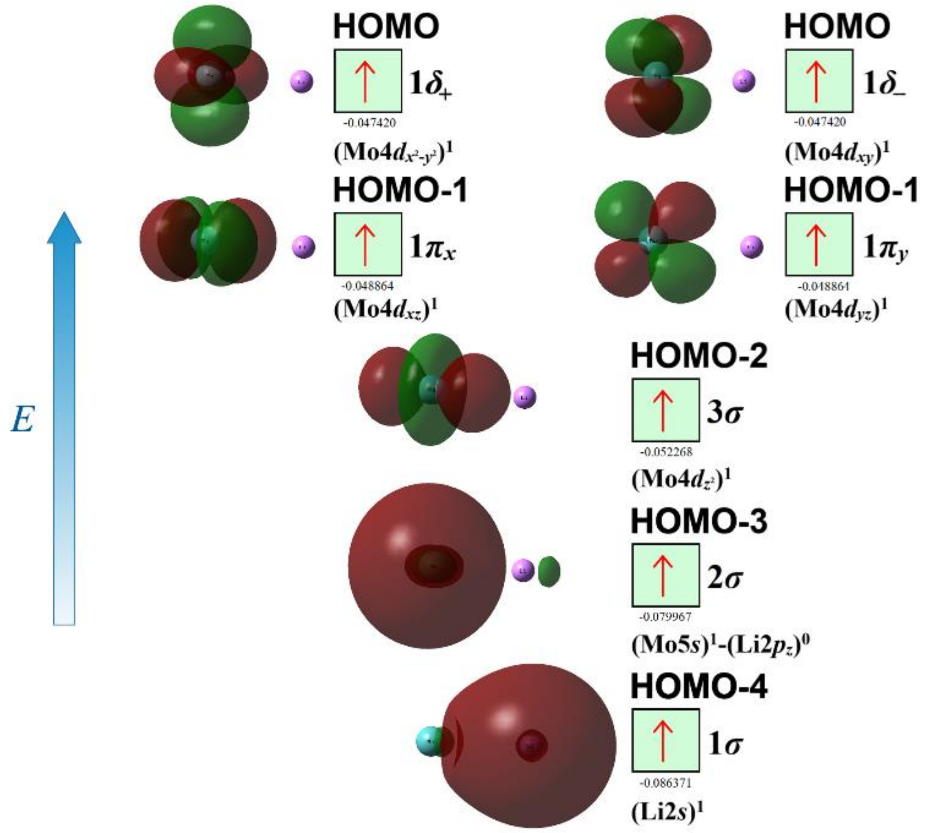

SCHEME S2: MOs energy diagrams of the excited state $^8\Sigma^+(1)$ of MoLi at the CASSCF/AV5Z level.

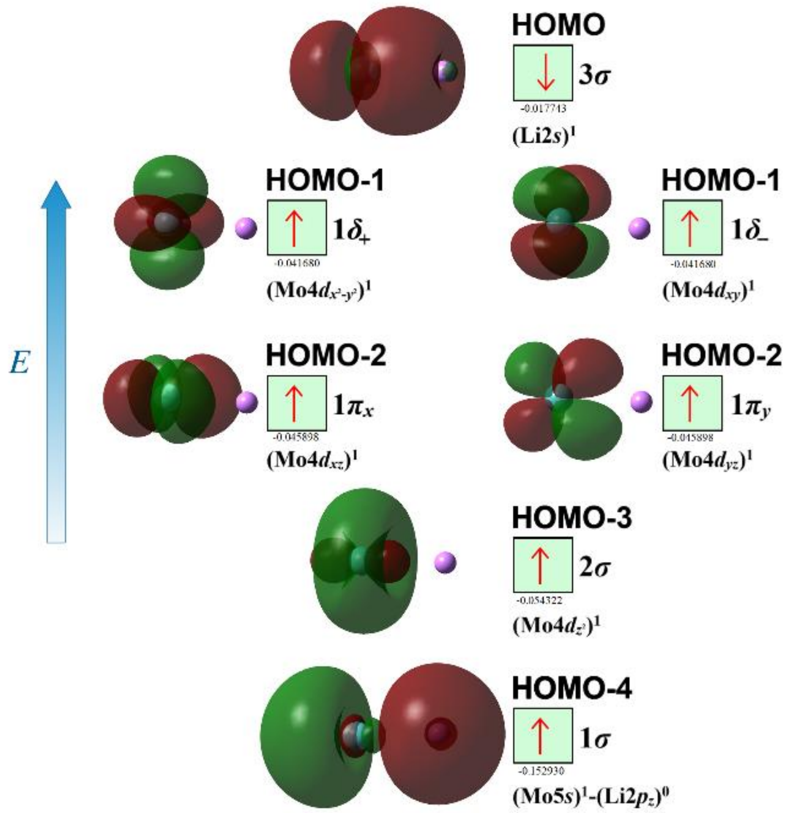

SCHEME S3: MOs energy diagrams of the excited state $^6\Sigma^+(2)$ of MoLi at the CASSCF/AV5Z level.

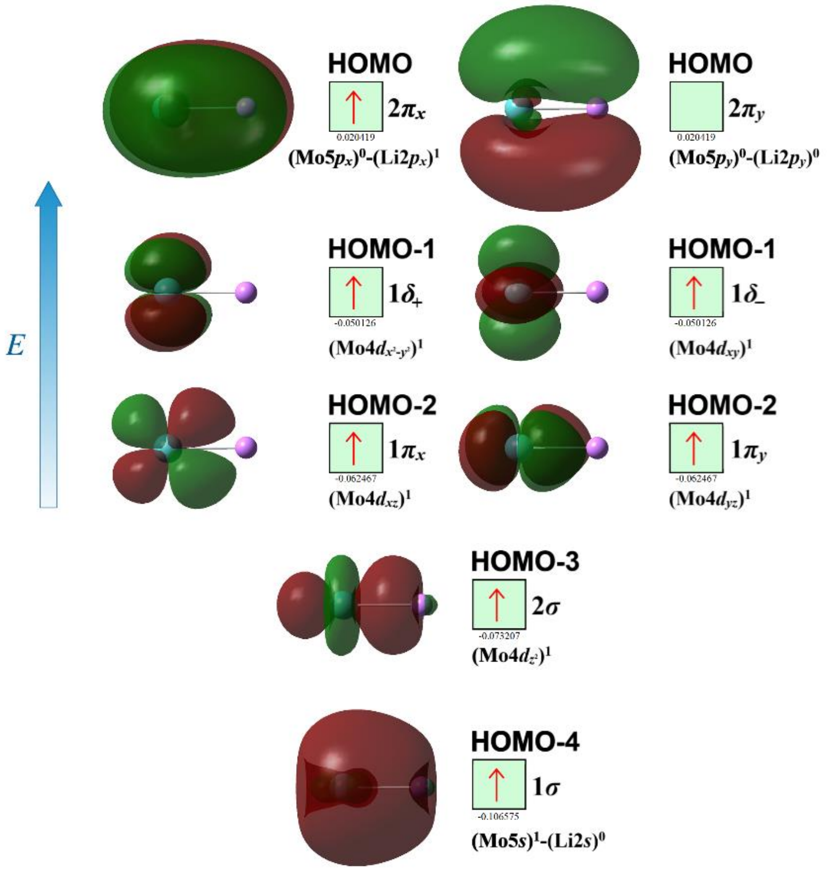

SCHEME S4: MOs energy diagrams of the excited state $^8\Pi(1)$ of MoLi at the CASSCF/AV5Z level.

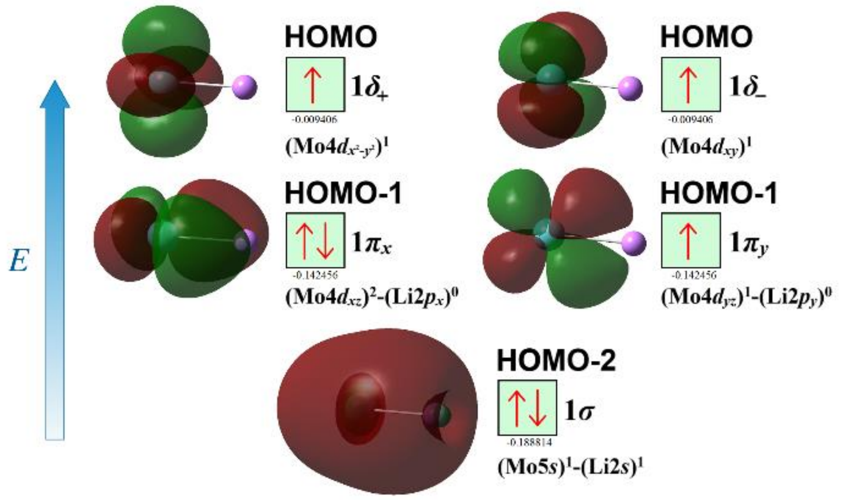

SCHEME S5: MOs energy diagrams of the excited state $^4\Pi(1)$ of MoLi at the CASSCF/AV5Z level.

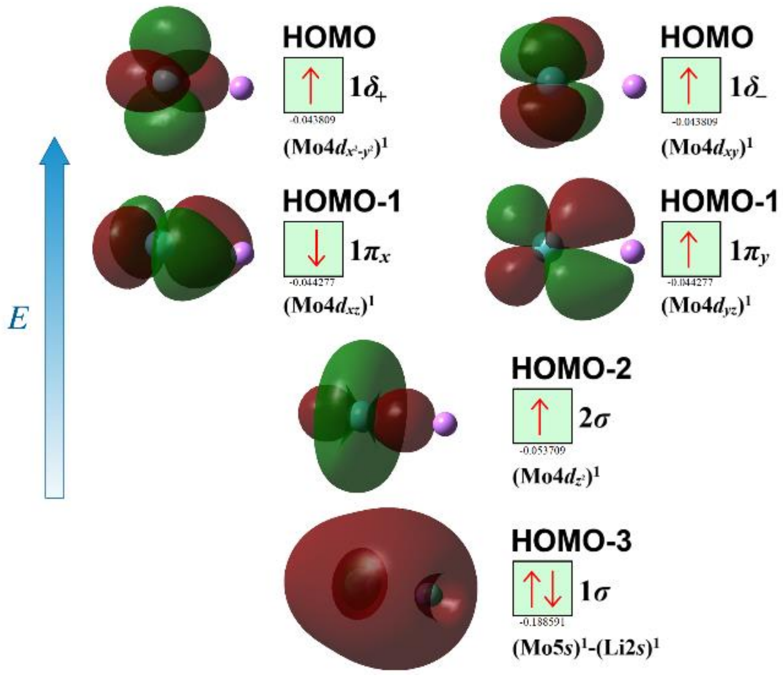

SCHEME S6: MOs energy diagrams of the excited state $^4\Sigma^+(1)$ of MoLi at the CASSCF/AV5Z level.

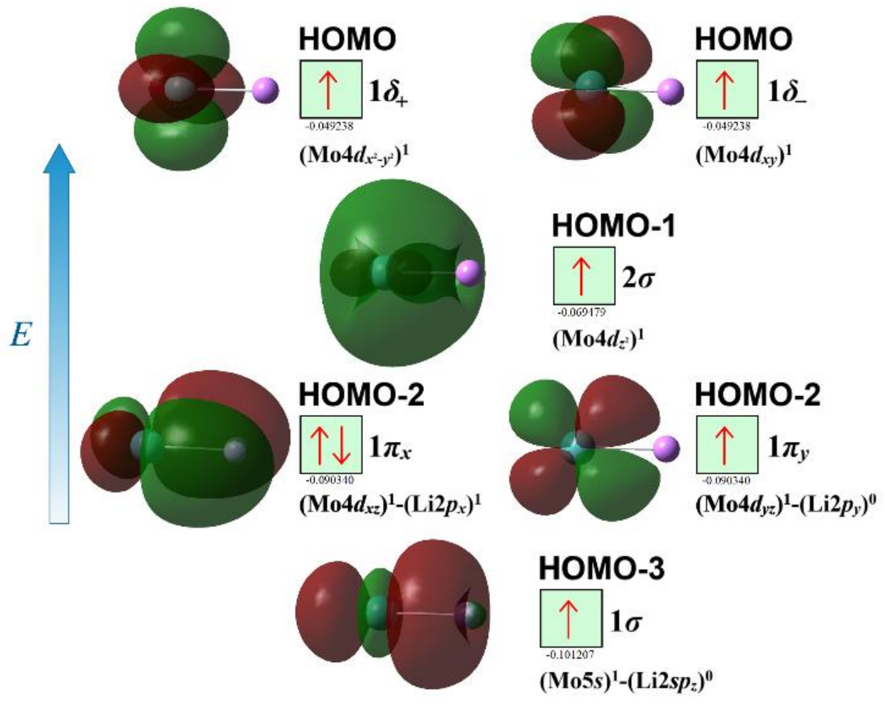

SCHEME S7: MOs energy diagrams of the excited state $^6\Pi(1)$ of MoLi at the CASSCF/AV5Z level.

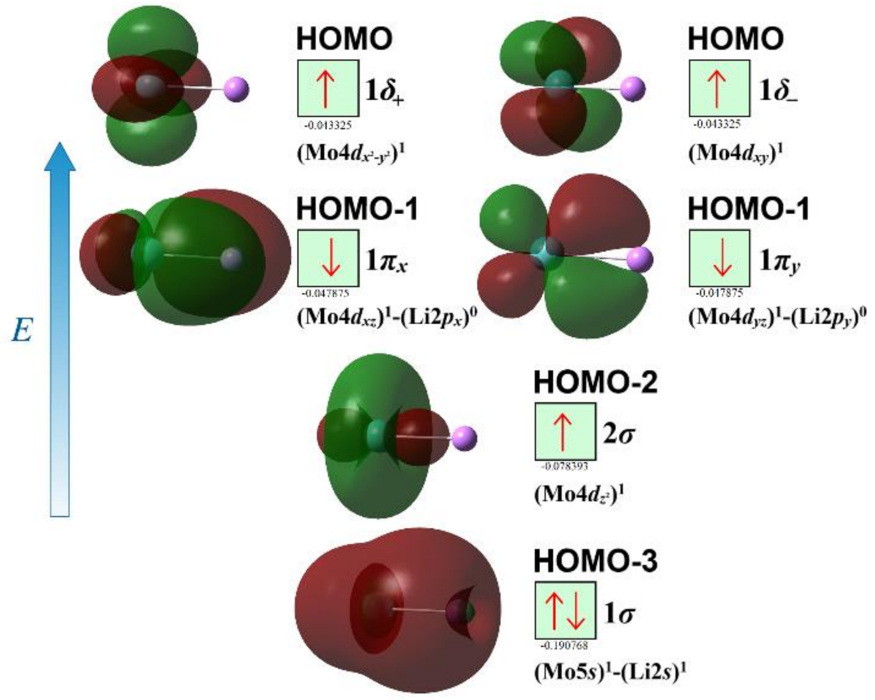

SCHEME S8: MOs energy diagrams of the excited state $^2\Sigma^+(1)$ of MoLi at the CASSCF/AV5Z level.

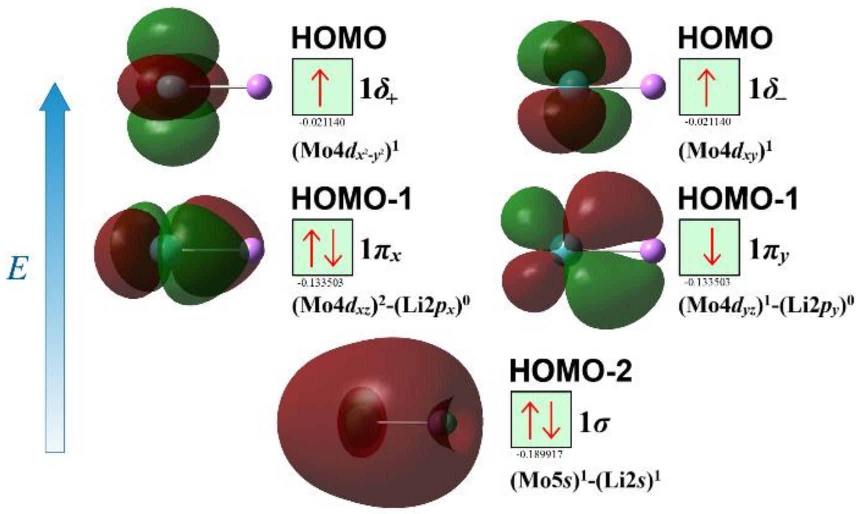

SCHEME S9: MOs energy diagrams of the excited state $^2\Pi(1)$ of MoLi at the CASSCF/AV5Z level.

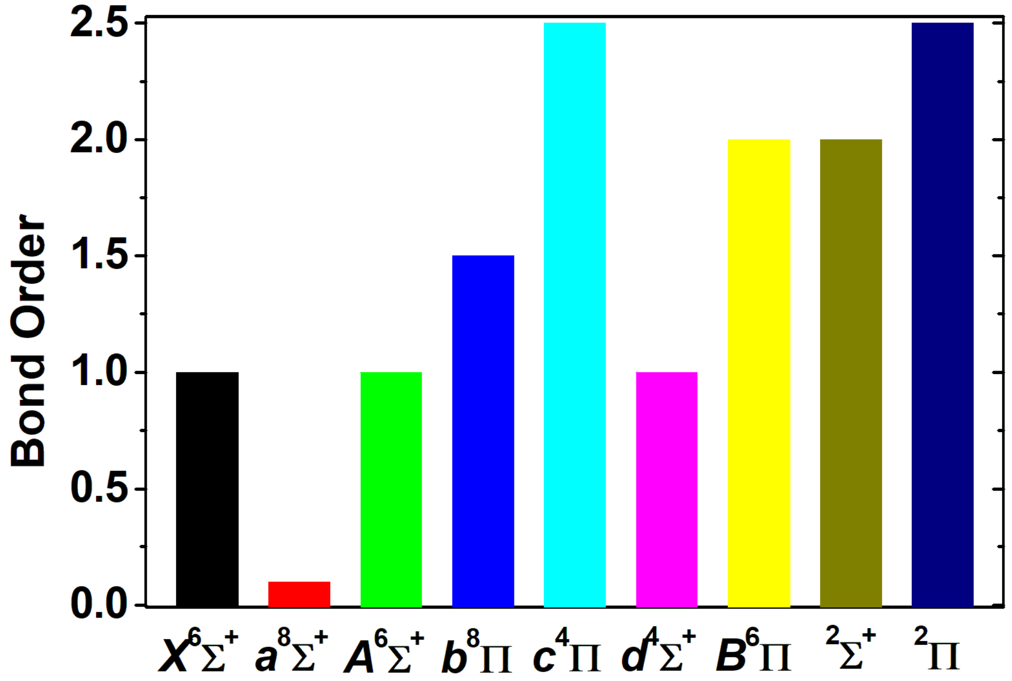

FIGURE S2: Bond order magnitude of selected states of MoLi at the MRCI(Q)/AV5Z levels.

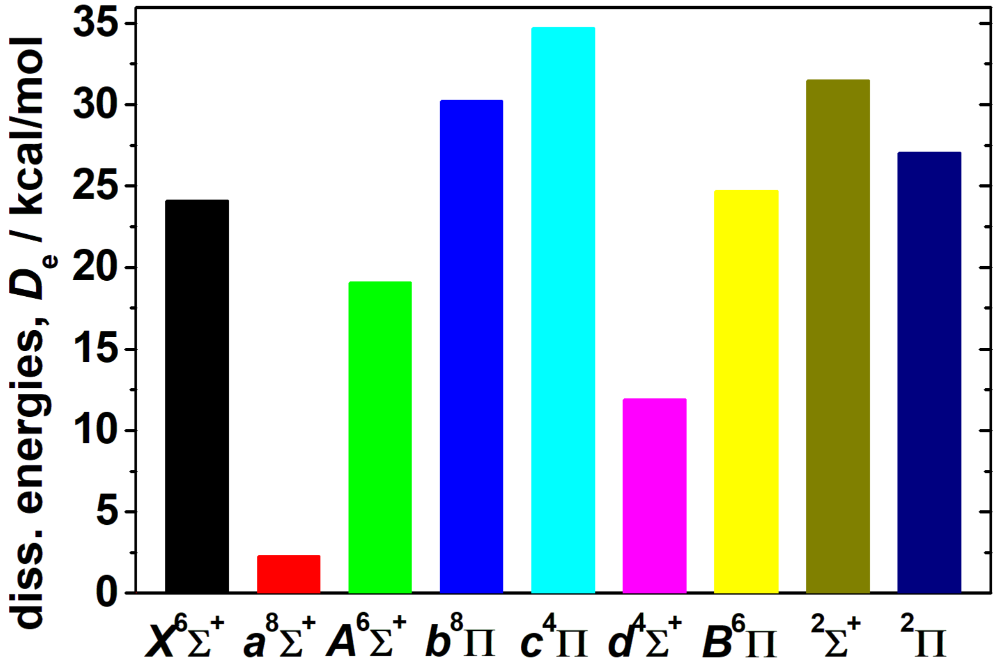

FIGURE S3: Dissociation energies of selected states of MoLi at the MRCI+Q/AV5Z level.

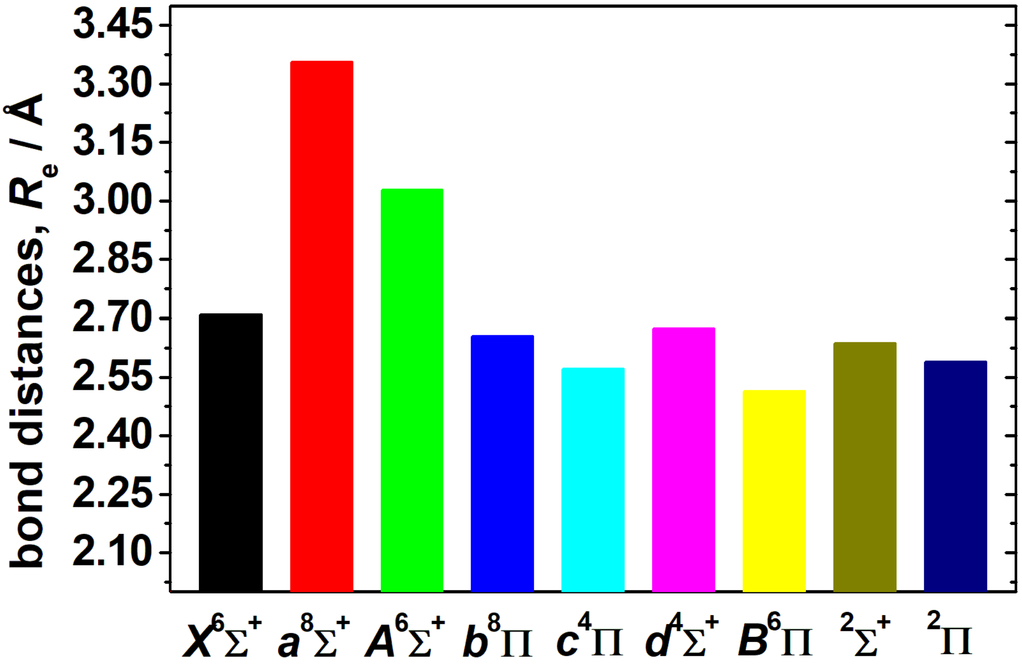

FIGURE S4: Bond distances of selected states of MoLi at the MRCI+Q/AV5Z level.

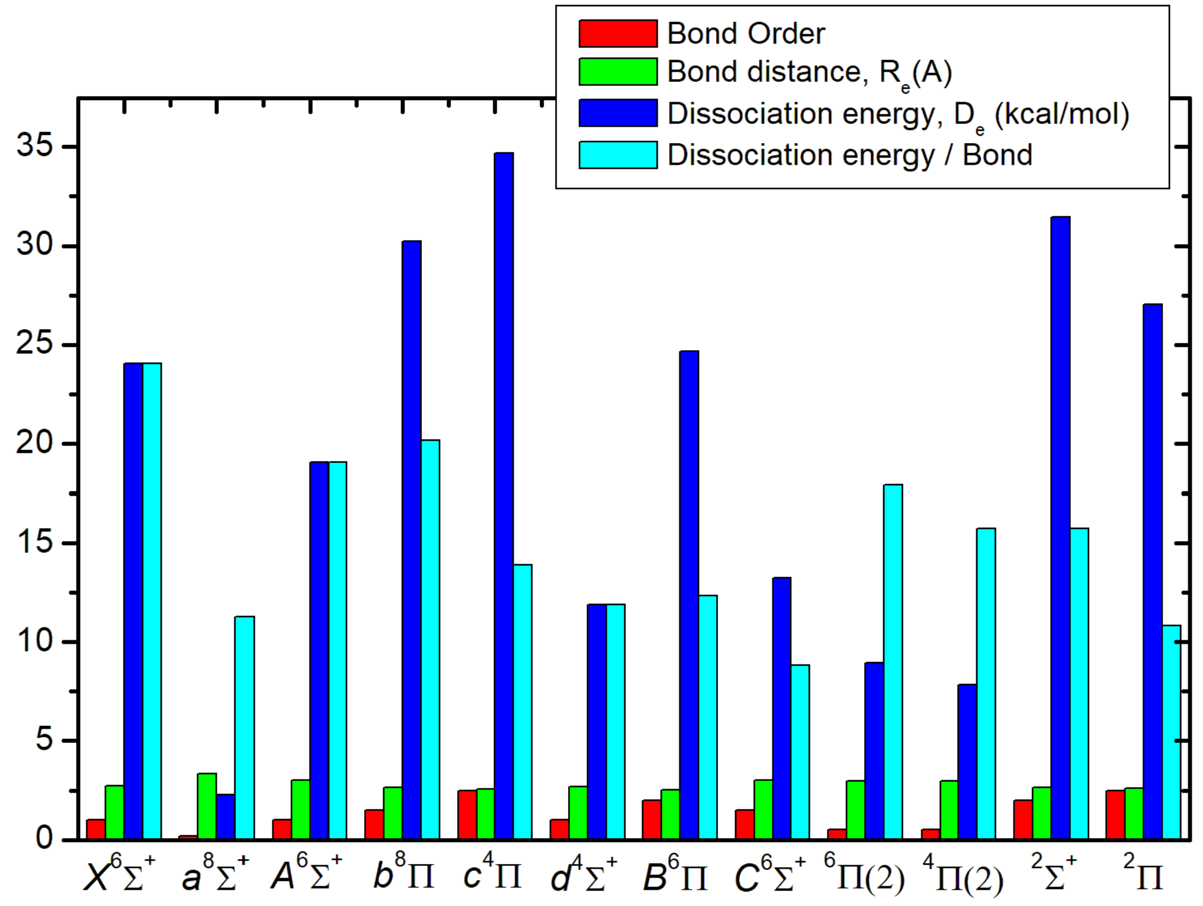

FIGURE S5: Bond order magnitude, bond distances, diabatic dissociation energies and dissociation energies/bond of 12 calculated states of MoLi at the MRCISD+Q/aug-cc-pV5Z(-PP) level.
